# An Imaging‐Guided Neural Model Explains Lexical Stress Alteration in Acquired Apraxia of Speech

**DOI:** 10.1002/hbm.70412

**Published:** 2025-12-11

**Authors:** Oren Civier, Amy Ramage, Jason Tourville, Donald A. Robin, Frank H. Guenther, Kirrie J. Ballard

**Affiliations:** ^1^ Swinburne University of Technology Melbourne Victoria Australia; ^2^ Australian National Imaging Facility Swinburne Node Melbourne Victoria Australia; ^3^ Department of Communication Sciences and Disorders University of New Hampshire Durham New Hampshire USA; ^4^ Department of Speech, Language, and Hearing Sciences Boston University Boston Massachusetts USA; ^5^ Interdisciplinary Program in Neuroscience and Behavior University of New Hampshire Durham New Hampshire USA; ^6^ Department of Biomedical Engineering Boston University Boston Massachusetts USA; ^7^ The Picower Institute for Learning and Memory Massachusetts Institute of Technology Cambridge Massachusetts USA; ^8^ Faculty of Medicine and Health The University of Sydney Camperdown New South Wales Australia; ^9^ The Brain and Mind Centre, The University of Sydney Camperdown New South Wales Australia

**Keywords:** apraxia of speech, DIVA, functional MRI, lexical stress, prosody

## Abstract

Acquired apraxia of speech (AOS) is a disorder of speech motor planning/programming that is induced by a lesion to the left anterior ventral precentral sulcus. This study analyses neuroimaging data from AOS in order to propose and computationally test a mechanistic explanation of how the lesion leads to the characteristic of altered lexical stress in the disorder. Neuroimaging data from 31 participants with left hemisphere stroke (15 AOS) were reanalysed to guide a ‘lesioned’ version of the bilateral GODIVA neuro‐computational model of speech production. Structural MRI and resting‐state functional MRI measurements were used to decide the model's lesion extent and atypical neural processing, respectively. The ‘lesioned’ model was compared with a neurotypical model on the production of an exemplar utterance with different linguistic contexts. Analyses revealed the average lesion in the AOS participants extended over 22.25% of the left anterior ventral precentral sulcus. Functional connectivity in AOS was reduced between the lateral part of that region and the right motor cortex, as well as between the left and right motor cortices themselves. The version of the model that we altered in line with these findings produced lengthening of the second of two consecutive short syllables. The lengthened syllable was a word‐initial unstressed syllable, and consequently, its contrastiveness with the adjacent stressed syllable of the word was reduced. The agreement between simulation results and previously reported acoustic measurements from actual AOS patients lends support to our mechanistic explanation. In conclusion, simulations of the GODIVA model provided empirical support for a mechanistic explanation indicating permanent sub‐threshold cortical activity in AOS. As a result, the speech system becomes biased away from a motor control strategy based on motor programs and toward a strategy based on sensory feedback. This both lengthens brief syllables and interferes with the mechanism to shift between syllables, ultimately altering lexical stress. Analysis of the model's neural dynamics suggests the explanation can be generalised to various contexts where lexical stress is altered in AOS.

## Graphical abstract

This study reanalysed neuroimaging data from individuals with acquired apraxia of speech (AOS) to simulate lesion effects in the GODIVA neurocomputational speech model. The lesioned model reproduced characteristic lexical stress alterations in AOS, supporting a mechanistic explanation of the disorder involving an engaged feedback control system and altered transitions between syllables.

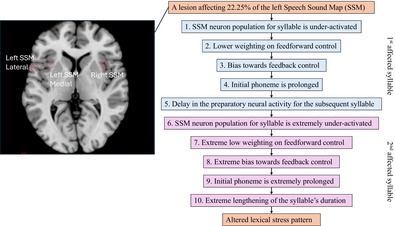

## 1 Introduction

Acquired apraxia of speech (AOS) is a brain lesion‐induced speech disorder characterized by several behavioural features including increased segment and intersegment durations, distorted phones, and more equal stress over words and/or sentences (Duffy 2013; McNeil et al. 2009). There is now some consensus on the lesions that give rise to the disorder (for a review, see Ballard et al. 2014). However, mechanistic explanations of how the structural damage leads to the behavioural dysfunction are largely lacking. The goal of this paper is to present a novel mechanistic explanation of the atypical neural dynamics that give rise to AOS, and to simulate them numerically using a neuro‐computational model of speech production (i.e., a custom instantiation of the DIVA/GODIVA family of speech production models, see Bohland et al. 2010; Guenther et al. 2006). We use this modelling approach primarily to test the plausibility of the mechanistic explanation, but also to fine‐tune and make it more specific.

Most of the research on AOS has dealt with lesion location and behavioural traits. In recent years, it has become clear that the area damaged is the anterior part of the left lateral/ventral precentral sulcus, encompassing the ventral premotor cortex (BA6) and the anteriorly adjacent posterior inferior frontal gyrus (BA44). Being based on evidence from multiple imaging techniques (Hillis et al. 2004; New et al. 2015; Richardson et al. 2012; Whitwell et al. 2013), this realization has quickly gained broad acceptance. In terms of behaviour, while several features are associated with the condition, there has been some debate on the core feature or set of features necessary for reliable diagnosis. Ballard and colleagues have shown that reduced contrastiveness of lexical stress in polysyllabic words with weak stress on the initial syllable (e.g., banana: /bə’nana/) is a powerful predictor of AOS presence in stress‐timed English dialects (Ballard et al. 2016, 2014; Basilakos et al. 2017; Duffy et al. 2017; Haley and Jacks 2019; Landin‐Romero et al. 2021; Vergis et al. 2014). This trait likely emerges from disproportionately long durations of the word‐initial unstressed syllable (Vergis et al. 2014): the contrast between weakly and strongly stressed syllables within a word is often achieved (at least in part) by the former being short and the latter long (Klatt 1976), and in these cases, the contrast is reduced when the weakly stressed syllable is longer. Although AOS has additional key manifestations (Ballard et al. 2016; Basilakos et al. 2017; McNeil et al. 2016), we focus here on developing an explanatory mechanism for this robust lexical stress effect.

From the few mechanistic explanations of AOS that link lesion to resulting behaviour, perhaps the most popular is the defective motor program hypothesis (e.g., Guenther 2016). The hypothesis holds that the left BA6/44 contains neuronal ensembles that read out motor programs (also termed ‘mental syllabary,’ see Levelt et al. 1999; Levelt and Wheeldon 1994) in a *feedforward* fashion, that is, the motor program's commands are read out sequentially, and without consulting sensory *feedback*. Given this proposed function of the left BA6/44, the hypothesis further postulates that lesions to this region directly lead to distorted speech. However, this explanation does not readily account for the lengthening of syllables. A more plausible explanation therefore is an atypical weighting of motor control strategies, specifically a bias away from feedforward motor control (i.e., motor program‐based) and towards feedback motor control (Ballard and Robin 2007). This entails inherent delays, first because the signal that carries the motor program's commands to the muscles is dampened, leading to movements that take more time to complete, and second, because planning movements based on sensory feedback requires waiting for auditory/tactile feedback to be perceived and processed, which is a slow process (cf. Civier et al. 2010 study on stuttering)—see Tilsen's work for empirical support and a computational model of this effect (Tilsen 2022, 2024). In turn, such delays in motor execution might lead to prolongations of speech sounds (see fig. 4 of Civier et al. 2010).

**FIGURE 1:**
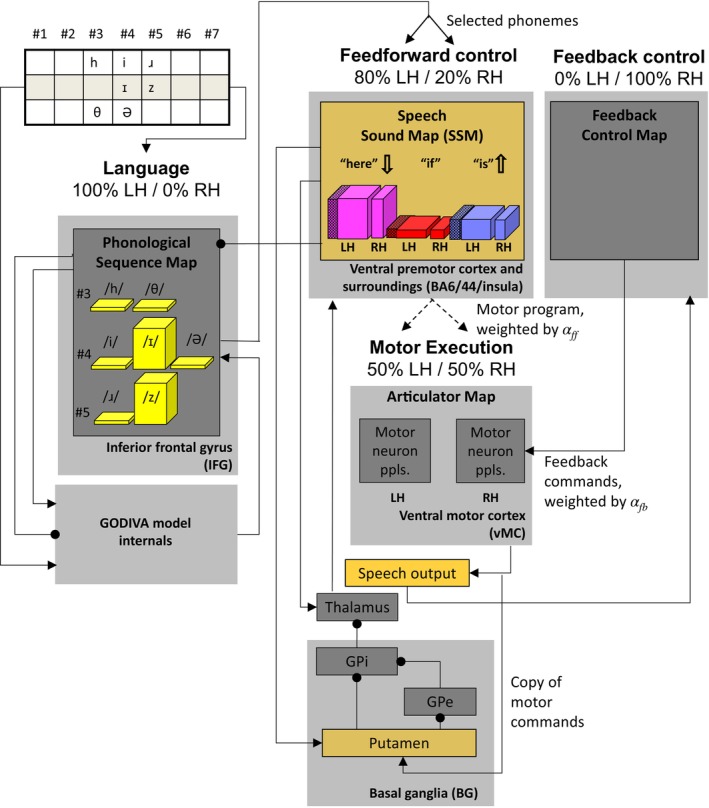
‘Box‐and‐arrow’ schematic of the bilateral GODIVA model. Shown is the bilateral GODIVA model amid a simulation representing a neurotypical brain (non‐AOS simulation). The model is divided into four interconnected systems: Language, Feedforward control, Feedback control and Motor Execution. The Feedforward and Feedback control systems, as well as the Basal Ganglia component of the motor execution system are described in Supporting Information. Full model description is available in Bohland et al. (2010) and Golfinopoulos et al. (2010). The input to the model is the utterance ‘here is the,’ encoded as the position‐specific phonemes that constitute the utterance (top‐left corner) and the audible output is represented by the speech output box (centre). Notice that the speech output feeds back into the Feedback Control, which uses external information to control speech based on current circumstances. This is different from feedforward control, which uses solely internal information (pre‐planned speech). Neuron populations are represented by bars, which are split into left hemisphere (LH) and right hemisphere (RH) populations. Shown is the point in time (asterisk in Figure 5a) when the speech system is executing the syllable ‘here’ and is preparing to shift to the syllable ‘is’; accordingly, the neuron population for the ‘here’ motor program has the highest activity in the SSM (tallest bar) but is decreasing (thick arrow pointing down), whereas the population for the ‘is’ motor program has low activity that is increasing (short bar and up‐pointing arrow). Also shown is the neuron population for ‘if’; it is weakly excited because, unlike ‘is’, it is only a partial match to the /I/ and /z/ phonemes currently active in IFG. The three boxes, whose activity/output is plotted in Figure 5a–c, are coloured in beige. For simplicity, details of some of the model's internal circuitry are omitted, and cortical regions are represented by their ‘choice’ layer only (see Supporting Information for more details). Thin arrows indicate projections between regions, either excitatory (normal arrowhead) or inhibitory (circle). For convenience, we also mark the model components that would be affected in an AOS simulation. Hatched segments on the bars representing SSM neuron populations show the portions ‘lesioned’ in AOS, while dashed arrows indicate the connections assumed to be weakened in AOS. GPi = internal globus pallidus. GPe = external globus pallidus. IFG = inferior frontal gyrus. SSM = speech sound map. Ppls. = populations. LH = left hemisphere. RH = right hemisphere.

Building upon past explanations, this paper presents a novel mechanistic explanation (or hypothesis) of how BA6/44 lesions give rise to context‐dependent lexical stress alteration in AOS. To test the plausibility of the explanation, we implement it in a computational model, run a simulation of the model, and then test the output of the model against empirical data. Our model of choice is the bilateral GODIVA, a variant of the extended GODIVA model previously utilized for studying developmental stuttering (Civier et al. 2013) (Figure 1; see Supporting Information for a detailed explanation of the bilateral GODIVA model). The presented model will join the many previous instantiations of the DIVA/GODIVA neuro‐computational models of speech production (Bohland et al. 2010; Civier et al. 2013; Golfinopoulos et al. 2010; Guenther 1995; Guenther et al. 2006, 1998; Kearney et al. 2020; Nieto‐Castanon et al. 2005; Tourville et al. 2008). An integral feature of the DIVA/GODIVA family of models is their ability to computationally simulate speech output (Bohland et al. 2010) and, in this work, we will examine the duration of syllables generated by the model, comparing them to previously published measurements from real participants (Vergis et al. 2014).

Owing to the DIVA/GODIVA models' ability to simulate speech output, they can readily incorporate feedback mechanisms, including interplay between feedback control and higher‐level speech processing. Usually, the feedback control system of the model continuously monitors the speech output that was generated by the feedforward control system (see connection between speech output and feedback control in Figure 1), and kicks in only when needed (e.g., if speech is disrupted). In AOS, however, we assume that feedback control plays a more prominent role in compensating for the impairment in the feedforward control system. In practice, the model is designed such that the weightings on feedback and feedforward control (denoted *α*_*fb*_ and *α*_*ff*_, respectively) fluctuate based on neural dynamics, ultimately contributing to the contextual effects observed in AOS. A more detailed description of the feedback control system is provided in the Supporting Information.

An important aspect of this work is the use of neuroimaging to guide our mechanistic explanation rather than basing this explanation on theory alone. To that end, we first re‐analyse imaging data from a sample of adults with stroke‐related AOS previously reported by New et al. (2015) and, after evaluating the results, we present a mechanistic explanation that aligns with them. Our participants with AOS were able to approximate the production of target words, which is common in mild and moderate cases. Consistent with this, they demonstrated just partial destruction of neuron populations in BA6/44 that encode motor programs.

**FIGURE 2:**
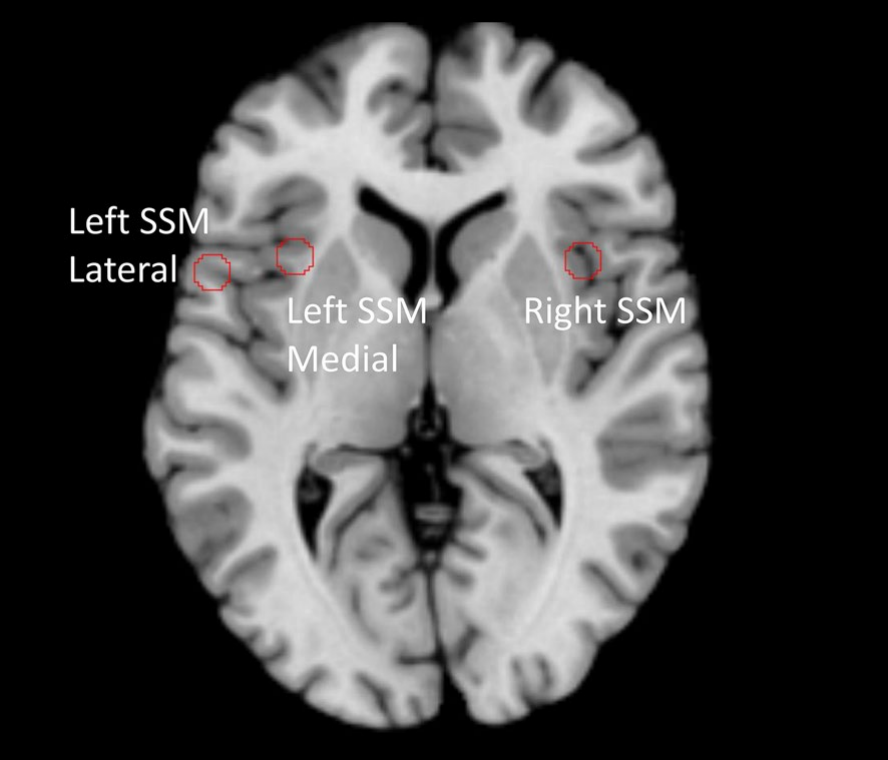
Brain region corresponding to DIVA/GODIVA speech sound maps (SSM). In the model, each well‐learned syllable has a motor program representation distributed across the left hemisphere and right hemisphere SSMs (see Figure 1). Brain regions are indicated by ROIs with a 5 mm radius.

In the DIVA/GODIVA family of models, model components are mapped to regions of interest (ROIs) in the brain. We take advantage of this important feature by performing the re‐analysis of the neuroimaging data using these ROIs, which then enables us to formulate the mechanistic explanation directly within the GODIVA framework. For example, based on accumulating evidence that syllable motor programs are encoded by bilateral, yet left‐dominant, neuron populations (Guenther 2016), the GODIVA model has left and right speech sound map (SSM) components, with the left one being mapped to both BA6/44 (lateral part) and left anterior insula (medial part) (see Figure 2, Table 1). As such, both BA6/44 and left anterior insula lesions are considered when calculating the left SSM ‘lesion’ in the model.

**TABLE 1:** DIVA/GODIVA components and corresponding brain region coordinates.

Feedforward control system	Feedback control system	DIVA/GODIVA component	Brain region	MNI coordinates
✓		Left SSM lateral	Left ventral BA6/posterior BA44	−55 9 0
✓		Left SSM medial	Left anterior Insula	−34 13 4
✓		Left Articulator Map	Left ventral Motor Cortex	−58 1 23
	✓	Feedback Control Map	Right Premotor Cortex	50 8 13
✓		Right SSM	Right anterior Insula	39 12 0
✓	✓	Right Articulator Map	Right ventral Motor Cortex	58 1 23

First, we present a neuroimaging experiment (Section 2) that re‐analyses the structural and functional MRI data from a previous study by our group (New et al. 2015). The results of the re‐analysis (lesion extent, functional connectivity alterations) are used in the modelling experiment (Section 3) to present a mechanistic explanation of AOS, which we then simulate using the bilateral GODIVA model. We run a detailed simulation of a single exemplar utterance, and to evaluate our mechanistic explanation, we test whether the alterations in syllable durations agree with previous literature. We wrap up by generalising our results and discussing their implications for AOS diagnosis and treatment.

## 2 Neuroimaging Experiment: Re‐Analysis of Structural and Functional MRI Data to Guide a Mechanistic Explanation of AOS

### 2.1 Introduction

In this experiment, we re‐analyse two imaging modalities (structural MRI and resting‐state fMRI) from New et al. (2015), a study that examined participants with AOS considered to have problems in motor program activation. The goal of the re‐analysis is to guide a mechanistic explanation of AOS: from the structural MRI we will extract the average extent of lesion, and from the resting‐state fMRI, the functional connections that are significantly affected in the disorder.

Because the mechanistic explanation will be tested using the GODIVA model, we used ROIs centred on the coordinates corresponding to the model's components (see Table 1). These ROIs differ from the ROIs used in the original analysis conducted in the New et al. paper, where the GODIVA model was not considered (see Eickhoff et al. 2009). Despite the role of subcortical structures in the model, the current imaging analysis is cortical‐only; the cortex has larger structures with mostly excitatory connections between them—compared with the smaller structures and inhibitory connections of the basal ganglia—and thus is easier to investigate with imaging.

Considering that pure cases of AOS are rare (e.g., Graff‐Radford et al. 2014), New et al. (2015) included data from adults that had AOS with aphasia (denoted here AOS‐plus‐aphasia). To isolate the traits of AOS, we also re‐analysed patients from New et al. that only had aphasia (denoted here aphasia‐only) and used them as a control group where warranted.

### 2.2 Methods

#### 2.2.1 Participants

Thirty‐one patients (15 AOS‐plus‐aphasia and 16 aphasia‐only) that were originally recruited for Ballard et al. (2016) contributed MRI imaging data. The MRI data were reported originally by New et al. (2015) and are here re‐analysed. The individual participant characteristics are shown in Table 2. The table also includes within‐group distribution of measures, as well as the statistics to compare the groups. Note that according to the table, our AOS population was assessed as having a predominantly apraxic motor speech impairment, that is, if a concomitant dysarthria was present, it was typically mild and less severe than the AOS (see Table 2, footnote e).

**TABLE 2:** Demographic and clinical data for participants included in the neuroimaging re‐analysis.

ID										WAB subtest scores					
	Sex	Age	MPO^d^	Edu	AOS severity	DYS severity	RCPM^d^	WAB AQ^d^	Aphasia type	Fluency	Spont. speech	Auditory comp^d^	Repetition^d^	Word finding^d^	Reading^d^
APH_005^a^	M	67	58	13	1.0	1.0	35	86.4	AN	6	15	9.95	8.9	9.4	60
APH_006^a^	M	49	14	17	1.0	1.0	32	72.5	AN	7	12	9.65	9.0	5.6	58
APH_008^a^	M	71	16	11	1.0	2.5	32	91.6	AN	8	17	9.80	9.7	9.3	60
APH_016^a^	F	56	44	13	1.0	1.5	32	93.0	AN	9	19	9.00	8.6	9.9	60
APH_034^a^	M	71	11	13	1.0	1.0	25	66.6	TS	6	9	6.40	9.8	8.1	36
APH_037^a^	M	59	69	11	1.0	1.0	27	80.7	AN	6	14	8.65	8.9	8.8	41
APH_041^a^	F	64	121	17	2.0	1.0	29	54.5	BR	4	12	4.85	5.0	5.4	25
APH_045^a^	F	55	92	19	1.0	1.0	35	98.7	ND	10	20	9.95	9.4	10.0	60
APH_047^a^	M	61	3	13	1.0	1.0	35	88.9	AN	9	18	9.45	7.3	9.7	53
APH_052^a^	F	45	37	17	1.0	1.0	35	36.9	BR	4	7	6.25	1.8	3.4	32
APH_062^a^	F	74	5	21	1.0	1.0	35	96.0	ND	9	19	10.00	9.6	9.4	60
APH_007^a^, ^b^	M	69	52	13	1.0	1.0	19	86.0	AN	9	19	7.40	7.6	9.0	59
APH_011^a^, ^b^	M	70	17	13	1.5	1.5	28	73.7	CO	6	15	9.75	6.6	5.5	58
APH_014^a^, ^b^	M	58	10	15	1.0	1.0	34	68.3	WE	6	14	6.45	4.6	9.1	60
APH_017^a^, ^b^	M	73	26	17	1.0	1.0	31	50.0	BR	4	9	6.80	4.2	5.0	31
APH_025^a^, ^b^	M	66	21	16	1.5	2.0	27	97.3	ND	10	20	9.45	10.0	9.2	60
AOS + APH_009^a^	M	48	17	11	7.0	2.0	21	11.3	GL	1	3	1.75	0.5	0.4	6
AOS + APH_018^a^	M	69	27	19	3.0	2.5	34	80.8	AN	5	14	9.40	8.1	8.9	NA
AOS + APH_019^a^	M	72	156	11	2.5	3.0	26	81.3	AN	5	14	8.65	8.8	9.2	60
AOS + APH_027^a^	M	77	81	15	6.5	3.5	30	60.5	AN^c^	5	10	9.55	3.2	7.5	48
AOS + APH_030^a^	M	76	120	15	6.5	3.5	22	39.6	BR	2	5	9.30	3.3	2.2	38
AOS + APH_040^a^	M	50	9	15	5.0	4.0	NA	60.2	AN^c^	5	13	8.40	0.0	8.7	NA
AOS + APH_048^a^	M	75	36	15	6.0	3.0	15	17.8	GL	2	5	2.90	0.7	0.3	22
AOS + APH_055^a^	M	51	6	10	4.0	3.0	34	69.5	AN^c^	5	14	9.65	3.4	7.7	49
AOS + APH_058^a^	M	40	13	16	5.0	1.0	21	23.9	BR	2	2	7.45	1.7	0.8	14
AOS + APH_001^a^, ^b^	M	48	13	15	4.5	1.5	36	41.6	BR	2	5	7.40	2.6	5.8	26
AOS + APH_010^a^, ^b^	M	66	32	17	3.5	1.0	34	83.7	AN	8	15	9.85	8.9	8.1	49
AOS + APH_015^a^, ^b^	M	54	36	16	3.5	1.0	31	75.1	AN	8	15	8.15	8.8	5.6	60
AOS + APH_022^a^, ^b^	M	66	84	11	3.0	1.5	32	75.3	AN^c^	5	13	9.95	6.4	8.3	37
AOS + APH_043^a^, ^b^	M	63	23	19	2.5	1.0	30	62.8	BR	2	9	8.30	6.6	7.5	46
AOS + APH_049^a^, ^b^	M	57	1	11	4.5	1.0	28	64.8	TM	4	11	6.80	8.1	6.5	49
**APH Median**		**65**	**23.5**	**14**	**1**	**1** ^e^	**32**	**83.35**		**6.5**	**15**	**9.22**	**8.75**	**9.05**	**58.5**
APH IQR		12.75	40.25	4	0	0.12	7.25	24.07		3	7	3.05	3.25	3.83	20.25
**AOS + APH Median**		**63**	**27**	**15**	**4.5**	**2** ^e^	**30**	**62.8**		**5**	**11**	**8.4**	**3.4**	**7.5**	**46**
AOS + APH IQR		20	45.5	5	2.25	2	10.5	34.6		3	9	2.05	5.95	4.3	23
*p*		0.56	0.95	0.61	< 0.001	0.014	0.26	0.01^g^		—	—	—	—	—	—
Corrected *p* ^f^		—	—	—	—	—	—	—		< 0.01^g^	0.024^g^	1	0.036^g^	0.144	0.3

*Note:* In all cases except AOS and DYS Severity, higher scores represent less impairment. APH = aphasia‐only, AOS + APH = Apraxia of speech with aphasia; M = male, F = female; MPO = months post onset of stroke; Edu = education (in years); AOS Sev/DYS Sev = severity of AOS or dysarthria on a Likert scale 1 (*absent*) to 7 (*severe*); WAB = Western Aphasia Battery—Revised (Kertesz 2006); AQ = Aphasia Quotient, an index of aphasia severity 0 (*most severe*) to 100 (*absent*); Aphasia types are AN—Anomic, BR—Broca, CO—Conduction, GL—Global, TM—Transcortical Motor, TS—Transcortical Sensory, WE—Wernicke; ND indicates that aphasia was not detected by WAB (i.e., AQ > 93); RCPM = Raven's Colored Progressive Matrices out of 36; WAB subtests: Fluency out of 10, SpontSp = spontaneous speech out of 20, Aud Comp = auditory comprehension out of 10, Repetition out of 10, Word Finding out of 10, Read = reading out of 10; NA = not able to complete test. Median values are in bold.

^a^ Reported in New et al. (2015) and re‐analysed here; see Supporting Information for mapping of ID codes.

^b^ Reported in Vergis et al. (2014).

^c^ Four cases were classified as conduction aphasia due to scores < 7 on the repetition subtest. As AOS could explain all or part of their lower repetition performance, they were conservatively coded as anomic aphasia.

^d^ Mann–Whitney *U* test, two‐tailed, was used to assess group differences given non‐normal distribution of the variable.

^e^ Median dysarthria severity was significantly greater in the AOS + APH group, with a median of absent (1.0) in the APH group and minimal (2) in the AOS + APH group. Nevertheless, dysarthria was not treated as a confound as, in all but one AOS + APH case, the AOS was rated as more severe than the dysarthria, and for the remaining case, AOS was rated minimal–mild (2.5) and dysarthria as mild (3.0).

^f^ The *p* values of the WAB subtest scores' *t* tests were Bonferroni corrected for six comparisons (one comparison per subset).

^g^ Note that the AOS + APH group had significantly lower median scores (more impairment) than the APH group on WAB AQ, Fluency, Spontaneous Speech, and Repetition. This result must be interpreted with caution as the presence of AOS can impact these expressive tasks and resulting estimates of aphasia severity.

Participants were recruited from speech‐language pathologists and local and national support networks around metropolitan Sydney, NSW, Australia. Inclusion criteria included: speech‐language difficulty, single left hemisphere stroke, between 18 and 75 years of age, right‐handed per self‐report, premorbid fluency in English, no contraindication for MRI, and no history of cognitive impairment, premorbid speech‐language deficits, substance abuse, or uncorrected hearing, vision or other sensory impairment. Participants gave informed written consent using procedures approved by the human ethics committees of the Sydney Southwest Area Health Service and the University of Sydney.

Diagnosis of AOS was determined by consensus between two expert raters (see Ballard et al. 2016 for details). Briefly, a 15–20 min edited video was generated for each case that included assessment tasks of oral structure and function, sustained phonation, alternating and sequential motion rate (i.e., multiple repetitions of ‘papapa’ and ‘pataka,’ respectively), counting and saying the days of the week, single and multi‐syllabic word production, passage reading, and story‐retelling or conversation. The raters judged the presence/severity of AOS using a 7‐point Likert‐type scale (1 *absent*, 2 *minimal*, 3 *mild*, 4 *mild‐moderate*, 5 *moderate*, 6 *moderate‐severe*, 7 *severe*), applying criteria described by Duffy (2013) and McNeil et al. (2009). Disagreements on presence or severity were resolved by consensus with a third rater. More details on the participants can be found in New et al. (2015).

#### 2.2.2 MRI Data Acquisition

As reported in New et al. (2015), structural and functional MRI data were acquired on a Philips 3T TX scanner. High‐resolution T1‐weighted structural images were acquired for each participant at 1 mm^3^ resolution for spatial normalization and localizing functional effects. Resting‐state gradient‐echo, echo‐planar images were acquired using blood‐oxygen‐level‐dependent (BOLD) contrast using the following parameters: TR = 2.2 s, TE = 30 ms, flip angle = 90°, in‐plane resolution = 3.1 × 3.1 × 3.1 mm^2^ for 36 axial slices covering the entire brain, resulting in a total of 216 images. Participants were instructed to lie still, stay awake, and keep their eyes open for the duration of the scan.

#### 2.2.3 Structural Lesion Analysis

The structural T1 scan was spatially normalized to standard MNI space using the unified segmentation algorithm (Ashburner and Friston 2005) within SPM8 (Wellcome Trust Centre for Neuroimaging; http://www.fil.ion.ucl.ac.uk/spm). For the stroke patients, an additional empirically derived ‘lesion’ tissue class was added to segmentation priors to differentiate lesions from grey and white matter and CSF (Seghier et al. 2008). These output images were smoothed with an isotropic kernel of 8 mm at full width at half maximum. The value of each voxel in the smoothed image represented the probability that the tissue belonged to the assigned tissue class. The lesion tissue class image for each subject was analysed with the automated lesion identification algorithm (ALI toolbox, SPM8, Seghier et al. 2008) to quantify lesion volume. The percentage of lesioned tissue in the ‘left SSM lateral’ and ‘left SSM medial’ regions was calculated for each subject by placing a sphere with a 6 mm radius at the centre coordinates for these regions. Structural image analysis was performed on the AOS‐plus‐aphasia sample as this gave us the best estimate of the lesions in AOS, the condition at the focus of the simulation experiment that follows.

#### 2.2.4 Functional Connectivity Analysis

The first study that analysed the MRI images from our cohort (both the AOS‐plus‐aphasia and aphasia‐only patients) did not detect excessive head motion when compared to neurotypical controls (New et al. 2015), which permits us to perform resting‐state functional MRI analysis. Time courses were the first eigenvariate of the resting‐state signal for each of the peak coordinates of the ROIs using a sphere with a 5 mm radius (e.g., Figure 2).

Functional connectivity amongst the ROIs of interest was investigated in the AOS‐plus‐aphasia sample and the aphasia‐only sample (cf. Langner et al. 2015; Roski et al. 2013). Linear (Pearson) correlations between each of the seed ROI time series were conducted to examine the strength of connectivity. Correlations were then transformed into Fisher's *Z* values such that each score represents the inter‐regional functional connectivity strength. Comparisons between the AOS‐plus‐aphasia and the aphasia‐only groups were determined using independent samples *t*‐tests, thresholded at FDR‐corrected *p* < 0.05.

### 2.3 Results

#### 2.3.1 Extent of Lesions in Clinical Data

The average lesion extent in the left SSM lateral component in the AOS‐plus‐aphasia group was 20.03%. In the left SSM medial, it was 24.49%.

#### 2.3.2 Functional Connectivity in Clinical Data

**FIGURE 3:**
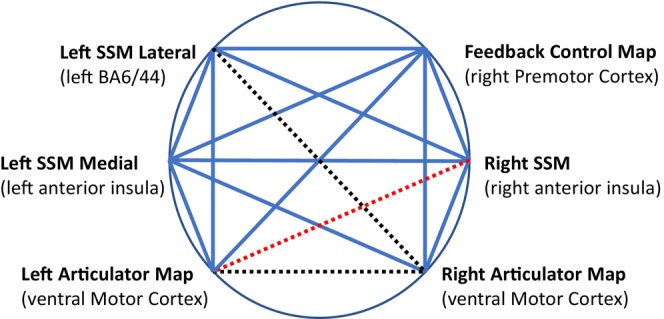
Functional connectivity data using the regions of the bilateral GODIVA model. Dashed lines indicate AOS‐plus‐aphasia < aphasia‐only, *p* < 0.05, FDR corrected. Red dashed lines indicate that, in addition, connectivity strength in the AOS‐plus‐aphasia group is negative. Blue lines indicate no group difference. See Table 1 for region details.

The results of the functional connectivity analysis (Figure 3) comparing aphasia‐only with AOS‐plus‐aphasia showed that the latter group had: (1) reduced connectivity between the left SSM lateral and the right articulator map (aphasia‐only *r* = 0.25 [SD = 0.25], AOS‐plus‐aphasia *r* = 0.01 [SD = 0.19]); (2) reduced connectivity between the left and right articulator maps (aphasia‐only *r* = 0.42 [SD = 0.32], AOS‐plus‐aphasia *r* = 0.11 [SD = 0.22]) and (3) reduced connectivity between the right SSM and the left articulator map (aphasia‐only *r* = 0.12 [SD = 0.25], AOS‐plus‐aphasia *r* = −0.09 [SD = 0.15]). All results were significant when corrected for multiple comparisons.

### 2.4 Discussion

In this section, we re‐analysed multi‐mod al MRI data from AOS‐plus‐aphasia and aphasia‐only patients, using the framework of the GODIVA model. The analysis of the results below will serve to guide a mechanistic explanation and simulations of AOS in the modelling experiment.

#### 2.4.1 Neuron Death in Left SSM

The results indicate that in AOS‐plus‐aphasia patients, the lesion spanning the two adjacent left SSM regions is substantial. Given our assumption that the two regions share the same functionality, we treat them as a single region. Therefore, our mechanistic explanation will assume that in the AOS population, the percentage of dead neurons in the unified left SSM region is 22.25% on average (the mean over left SSM lateral and medial).

#### 2.4.2 Degeneration of Projections From Left SSM to the Articulator Maps

The reduced functional connectivity between the left SSM lateral and right articulator map is consistent with the neural death in left SSM lateral in the AOS group. We assume that the affected neurons originally projected to the right articulator map and that their death led to the efferent degeneration. However, there was not a significant parallel reduction in functional connectivity between the left SSM medial and the right articulatory map. Nevertheless, for the mechanistic explanation, we assume that the left SSM medial–right articulator map connection includes degenerated projections as well, for the following reasons: (1) both left SSM medial and lateral had a similar extent of neural death, (2) left SSM lateral and medial share the same functionality, thus their neurons should similarly project to the right articulator map, (3) a trend in the data suggests that the AOS‐plus‐aphasia group may have reduced functional connectivity in this connection (*p* = 0.075, uncorrected) but the analysis suffered from lack of power.

To partially address the lack of power, as well as the heterogeneity in the sample (different types of aphasia within both groups), we performed a follow‐up analysis by substituting the aphasia‐only group (16 participants) with a larger group of neurotypical speakers (18 participants) who were included in the same original study (New et al. 2015). Based on the trend in our initial analysis, we then tested the hypothesis that AOS‐plus‐aphasia patients (same patients from the initial analysis: 15 males; age = 60.8 ± 11.9 years, education = 14.4 ± 2.9 years) have weaker connectivity than neurotypical controls (8 males, 10 females; age = 62.5 ± 8.8 years, education = 16.2 ± 4.1 years) in the left SSM medial—right articulator map connection. The null hypothesis was rejected with *p* = 0.0449, thus confirming our prediction. Together, the results of the initial and follow‐up analyses lend support for the degeneration of left SSM medial—right articulator map in AOS.

Based on anatomy, premotor neurons controlling speech (left SSM lateral and medial) should project to both the left and right articulator maps, so the finding that the left SSM neural death only affected connectivity with the right (not left) articulator map is surprising. As above, also here we compared the AOS‐plus‐aphasia group to the newly added control group, but no significant differences were detected in SSM connections with the left articulator maps. This could relate to artefacts arising from the smoothing applied in fMRI analysis and/or the warping that is required to register all participants to a common template. Both might cause the measurement of activity from one region to be contaminated by the surrounding regions, making the correlations between time courses of adjacent regions appear higher than they are. This might mask reductions in functional connectivity between adjacent regions, in this case, between the left SSM lateral/medial and the left articulator map. Assuming that the proximity artefact indeed masked reduced connectivity in the ipsilateral connections, the mechanistic explanation suggests weak connectivity from the lesioned unified left SSM to both the left (ipsilateral) and right (contralateral) articulator maps. The general discussion suggests alternative interpretations in case future studies will prove the assumption wrong (see Section 4).

#### 2.4.3 Engagement of the Feedback Control System in the Right Hemisphere

As in Section 2.4.2, the reduced functional connectivity between left and right articulator maps in AOS might reflect weaker structural connectivity. However, we suggest that the reduced functional connectivity should be sourced instead to the left and right articulator maps performing different processing tasks. After all, functional connectivity between two brain regions is defined as the correlation between their corresponding activity time courses, and such correlation would be reduced if the regions diverge in their roles.

Recall that only the right articulator map belongs to the feedback system (Table 1). In the neurotypical brain, the feedback system is barely engaged; thus both left and right articulator maps act the same as part of the feedforward control system. In AOS, however, we suggest that the feedback control system is strongly engaged; thus generating in the right articulator map a different pattern of activity than in its left counterpart; hence, reduced functional connectivity between them. This is definitely valid during speech and, due to the AOS condition being chronic, should also leave its mark on the resting‐state activity we examine here.

To support our suggestion that the feedback control map is more strongly engaged in AOS, one might also expect stronger functional connectivity within the feedback control system, namely between the feedback control map and the right articulator map. However, such an effect was not detected in our analysis. Although surprising, the lack of effect may be corroborated by a previous study where reliance on feedback control was artificially induced in fluent speakers (Tourville et al. 2008). Also there, no increase was noted in the functional connectivity between the two aforementioned regions. A more direct way to examine the engagement of feedback control in AOS is to measure regional activities during speech, rather than connectivity at rest, but such studies are lacking. That said, one brain stimulation case study on AOS does provide indirect evidence for the engagement of the right precentral gyrus, which includes the feedback control system's right articulator map (Kaufmann et al. 2019).

#### 2.4.4 Negative Functional Connectivity Will Not Be Modelled

Because there is a controversy regarding the meaning of negative functional connectivity (Mehler and Kording 2018) and it might be an artefact of the global signal regression of the mean we applied (New et al. 2015), the negative correlation in AOS‐plus‐aphasia between the right SSM and left articulator map (and the significant difference from the aphasia‐only) will not be considered in the mechanistic explanation. To corroborate this result, future studies of AOS should measure resting‐state functional connectivity using techniques that do not require global signal regression (e.g., electroencephalography, magnetoencephalography, functional near‐infrared spectroscopy).

If future studies find the negative functional connectivity genuine, this may support inter‐regional inhibition, and drive a refinement of our model. For example, instead of assuming that lesions completely destroy neurons, they can only damage them. The neurons will keep reading out motor programs, but those are likely to be incorrect or noisy and lead to faulty feedforward control signals. The response of the CNS may be to try minimising the activity of the left motor cortex, which is solely driven by the faulty signals, while preserving activity in the right motor cortex (in contrast with left motor cortex, the right motor cortex is also driven by feedback control, which is still intact). To implement this, the CNS may recruit right SSM neuron populations to inhibit the left motor cortex. Indeed, inhibitory connections from right SSM to left motor cortex are consistent with stimulation studies in animals (Quessy et al. 2016).

## 3 Modelling Experiment: Simulating a Mechanistic Explanation of AOS Using the Bilateral GODIVA Model

### 3.1 Introduction

In this section, we take the conclusions of the neuroimaging experiment in Section 2, combine them with theoretical considerations, and present a detailed mechanistic explanation. Using the bilateral GODIVA model, we then test whether the explanation is plausible.

The results of the neuroimaging experiment call for a mechanistic explanation that includes neural death in the left SSM maps, as well as weakened connectivity from the left SSM maps to the bilateral articulator maps, and engagement of the right‐lateralised feedback control system. This resonates well with the previously suggested bias away from feedforward motor control and towards feedback motor control (see Section 1) and forms the basis of our mechanistic explanation. However, this explanation still lacks an element that can account for the context‐dependent pattern of syllable lengthening in AOS (Vergis et al. 2014). We suggest a mechanistic explanation that incorporates a time‐varying bias towards feedback control, with the extent of bias being modulated by momentary activity of SSM neurons. As such, in the AOS simulation, each syllable is being initiated with slightly different SSM neuron activity level (compare with Supporting Information, where activity must reach a specific threshold), with the exact level being modulated by neural dynamics during the execution of the previous syllable (see Sections 3.2.1 and 3.2.2 below). Our suggestion borrows from a similar approach applied to developmental stuttering (Civier et al. 2013) but with important differences: AOS, unlike developmental stuttering, involves a brain lesion in BA6/44 after speech production has matured.

Another element of our mechanistic explanation is the context‐dependent relation between the feedback‐reliance‐induced prolongation of speech sounds (see Section 1) and the lengthening of syllables. According to the bilateral GODIVA model, if a sound within a syllable is prolonged, the shift‐preparation trigger will be delayed, postponing the shift to the next syllable and thus lengthening the current one (see Supporting Information for the role of the basal ganglia in the model). In the AOS simulation, however, this process is modulated by the necessary introduction of alternative syllable‐shifting mechanisms (see Section 3.2.4 below): for certain syllables, sound prolongation will now lengthen syllable duration, whereas for others, syllable duration will remain unaffected. In the latter case, the sound prolongation will instead interfere with the preparation of the *next* syllable in line.

**FIGURE 4:**
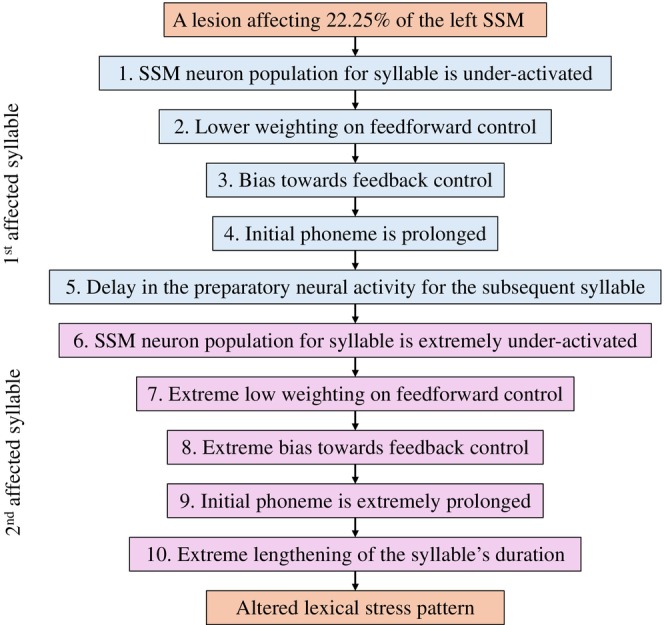
A mechanistic explanation of altered lexical stress in AOS. The box‐and‐arrow diagram describes the high‐level sequence of steps that leads from a lesion to altered lexical stress in the bilateral GODIVA model of AOS. The steps include changes in internal model variables as well as changes in model output, as these interact with each other. The steps are divided into two affected syllables: the first affected syllable is usually an unstressed monosyllabic word (e.g., ‘the’) and the second one is often a short word‐initial syllable (e.g., ‘po’ in ‘potato’). For time courses and comparison with a non‐AOS simulation, see Figure 5.

Combining the above elements, our mechanistic explanation is as follows (see Figure 4): a lesion affecting 22.25% of the left SSM, on average, causes neural populations for syllables to be always under‐activated. The reduced SSM activity translates to lower weighting on feedforward control (fewer active neurons are projecting to the articulator maps), biasing the speech production system towards feedback control. The bias towards feedback prolongs the initial phone of the ongoing syllable (see Section 1), which can, in turn, delay the preparatory neural activity for the next syllable (see previous paragraph). The next syllable starts then with an SSM neuron population that is even more under‐activated than the prior syllable, further increasing reliance on feedback control. This leads to extreme lengthening of the next syllable's duration and gives rise to an altered lexical stress pattern. This mechanistic explanation hypothesizes a domino effect, where altered production of one syllable amplifies the problem in the subsequent syllable.

We incorporate the mechanistic explanation in the bilateral GODIVA model to run an AOS simulation (representing an average AOS patient with motor program activation problems), and to test the plausibility of the explanation, we evaluate whether the simulation output—syllable durations—agrees with published acoustic measurements of syllable durations in AOS (Vergis et al. 2014). To facilitate comparison with Vergis et al., we simulate the same sentence structure used in that study, i.e., ‘here is the ____’. Because we are evaluating the tendency for AOS speakers to lengthen word‐initial unstressed syllables, we end the sentence with the word ‘potato’; one of the target words used by Vergis et al. The simulation is limited to a single utterance, but we closely examine the neural dynamics that lead to atypical speech output: to gain deeper understanding of the disorder, and to allow generalisation to other linguistic contexts. Lastly, to have a basis for comparison for the neural dynamics, we will also be running a non‐AOS simulation representing a neurotypical brain.

### 3.2 Methods

#### 3.2.1 Modelling Lesion in Left SSM: The Highest‐Active SSM Neuron Population Is the One Initiating the Syllable

In line with the mechanistic explanation, for the AOS simulation we will ‘lesion’ the model by eliminating 22.25% of the left SSM neurons in the AOS simulation (see hatches in Figure 1). Because 20% of the SSM neurons are in the right hemisphere, the elimination of 22.25% left‐hemisphere neurons represents 17.8% dead neurons in total. Under the assumption that SSM neurons are intermingled (see Supporting Information), all SSM neuron populations for the different syllables are equally affected. Notice that each population has its intact neurons distributed across the left and right hemispheres (Table 1), but because neural connectivity is the same bilaterally, we will simulate each population as a single unit for simplicity (i.e., the simulations do not make a distinction between left and right SSM, only between different neuron populations within the SSM). We note a previous model of language that simulated syllable formation from phonemes, and where the hemispheres were separate entities (Chang and Lambon Ralph 2020). We acknowledge that such an approach might be required to explain further observations on AOS such as plasticity‐related recovery but is outside the scope of the current investigation.

A loss of 17.8% of SSM neurons is predicted to permanently reduce activities of all SSM neuron populations, preventing them from reaching the threshold for reading out the motor program. This would predict no speech output in AOS, but here we follow Civier et al.'s (2013) suggestion that the speech system compensates for this potentially debilitating weakness by forcing motor program readout after all. It does this by selecting the highest‐active SSM neuron population and initiating its corresponding syllable, regardless.

#### 3.2.2 Modelling Weak Signal From SSM to the Articulator Maps: Lower Feedforward Control Weighting

When an SSM neuron population reaches threshold and reads out its motor program, readout will be normal (i.e., ‘normal‐readout threshold’). If the population does not reach threshold, the program's readout will be weak. The linear relationship between momentary SSM neuron population activity, *s*(*t*), and strength of motor program readout (i.e., weighting towards feedforward control), *α*_ff_, is represented in the GODIVA model using the formula (eq. 24 in the supporting information of Civier et al. 2013):

(1) $$\alpha_{\mathrm{ff}}=0.85\times \frac{s\left(t\right)}{T}$$

where *T* is the normal‐readout threshold, and the 0.85 constant is baseline *α*_ff_ (i.e., the weighting of feedforward control in the neurotypical brain; see Supporting Information). When *s*(*t*) equals *T*, the readout will be normal, when it is below *T*, the readout will be weak.

For the AOS case, we predicted that all SSM neuron populations would remain sub‐threshold throughout (*s*(*t*) *< T*) with weak readout of the motor program (*α*_ff_ < 0.85). Based on the discussion of the functional connectivity results, this weak readout will affect both the left and right articulator maps (see Section 2.4.2).

#### 3.2.3 Modelling Weak Feedforward Control: Kick‐In of Feedback Control

As *α*_ff_ will always be below 0.85 in the AOS simulation, *α*_fb_ will be above 0.15 (in the model, feedback control compensates for lack of feedforward control, so they always sum to 1, see Civier et al. 2010). This is corroborated by the imaging experiment, which suggests that the feedback control system is likely engaged in AOS to a greater extent than in non‐AOS (see Section 2.4.3). The reduced feedforward control signal together with the increased reliance on feedback control tends to prolong speech sounds in the DIVA/GODIVA model (Civier et al. 2010 and Section 1). Given that *α*_fb_ is directly related to *α*_ff_, here we quantify the prolongation based on *α*_ff_ alone.

Rather than simulating the prolongation for each phone at each context, we will calculate a standard prolongation factor based on data from the word ‘bit’ that was simulated with DIVA in Civier et al. (2010). Civier et al. reported a 150% prolongation of the syllable‐initial phone /b/ with a 70% reduction in *α*_ff_ (i.e., from 0.85 to 0.25). We define the prolongation factor *P*_estimate_ as the ratio between the resulting behaviour—the change in the duration of ‘b’ from its normal duration—and the catalyst—the change in value of *α*_ff_ from its baseline when producing ‘b’—both expressed as percentages:

(2) $$P_{\text{estimate}}=\frac{\%\text{chang}e_{\text{duration}}^{"b"}}{\%\text{chang}e_{\alpha_{\mathrm{ff}}}^{"b"}}=\frac{150}{-70}=-2.142\left(\left.\frac{\text{phoneme duration change}}{\alpha_{\mathrm{ff}}\;\text{change}}\right)\right.$$

The value of *P*_estimate_ is negative because the directions of change are opposing, that is, decreased *α*_ff_ leads to increased phone duration.

Using *P*_estimate_ and the instantaneous change in *α*_ff_ (change from the 0.85 baseline), we can now calculate the change in duration for each syllable‐initial phone *x* in the AOS simulation:

(3) $$\%\text{chang}e_{\text{duration}}^{x}=P_{\text{estimate}}\times \%\text{chang}e_{\alpha_{\mathrm{ff}}}^{x}=-2.142\times \%\text{chang}e_{\alpha_{\mathrm{ff}}}^{x}$$

Although non‐initial phones might be prolonged as well, we do not simulate or estimate their duration because they are taking place too late in syllable production to have a major effect on syllable timing.

Finally, it should be acknowledged that phone prolongation due to dampened feedforward control may somewhat vary (see Civier et al. 2010). Yet here we chose a pragmatic approach and extrapolated results from a simulation of a single consonant in a specific context. The extrapolation can be made more accurate by considering not only the weighting of feedforward control but also the unique characteristics of each phone: original duration, the amplitude of movement, the rate of movement, the sounds that surround it, and so forth. However, we believe that this level of detail is unnecessary for the current study that aims to explain the main trends in existing behavioural data rather than generating exact predictions.

#### 3.2.4 Modelling Readout of Sub‐Threshold SSM Neuron Populations: Alternative Syllable Shift Timing Mechanisms

To enable speech output in the AOS simulation, we postulate that the speech system forces SSM neuron populations to initiate syllables despite being sub‐threshold. A challenge for the speech system is how to perform this task while preserving the correct timing of syllables in an utterance. Usually, the timing of shifting from one syllable to another is intrinsic, when the SSM neuron population reaches the normal‐readout threshold. However, with the sub‐threshold readout mechanism, the system is deprived of this critical cue. We propose three alternative mechanisms to enable syllable shifting. As this is a first step towards modelling these mechanisms, we have not yet assigned them to specific brain regions.

##### 3.2.4.1 Alternative Shift‐Timing I: Relative to Shift‐Preparation Trigger

Unlike the actual timing of syllable shifting, the shift‐preparation trigger that initiates the shift processes earlier in time is not dependent on SSM populations' output. Instead, it depends on the articulator map output (see ‘copy of motor commands’ in Figure 1 and Supporting Information), which is unaffected by the lesion in AOS. We hypothesise that the AOS‐lesioned speech system uses the intact shift‐preparation trigger for the first alternative shift‐timing mechanism. Specifically, the model will continuously monitor the putamen and, on detecting the occurrence of a shift‐preparation trigger, it will perform the syllable shift after a fixed time period automatically (i.e., without consulting SSM output).

To preserve the normal flow of speech production, the length of the fixed time period approximates the time it would take for neural dynamics to bring the activity of the next syllable's SSM neural population up to threshold in an unlesioned version of the model. In AOS patients, it is assumed that this timing was implicitly learned premorbidly.

##### 3.2.4.2 Alternative Shift‐Timing II: Based on Memory Trace of the Preceding Syllable Duration

The timing of the next syllable may also be based on the expected duration of the ongoing syllable. We suggest this is the case only for short syllables, when both the ongoing syllable is highly frequent in the language and the production of the syllable with the specific duration is frequent. High frequency means that the speech system has produced this syllable/duration combination many times in the past and there is a robust memory trace of its typical duration.

To implement this mechanism in the AOS simulation, we simply time the next syllable based on the duration that the ongoing syllable had in the non‐AOS simulation. We will apply it strictly to the syllable ‘the’ as this is the only short syllable in the simulated utterance that has a frequent syllable/duration combination. The other short syllable—‘po’ in potato—is frequent but the specific duration (i.e., where the vowel is reduced to a schwa in initial word position) is not common.

##### 3.2.4.3 Alternative Shift‐Timing III: Relative to Utterance Onset Signal

For the first syllable of an utterance, neither of the previous two mechanisms can be used. However, we assume that the adult speech system has an established memory trace of the typical latency from the utterance‐initiation signal (see Supporting Information) to when the first syllable is initiated, and this is used by the model to time the first syllable in the AOS simulation.

#### 3.2.5 Computational Simulations of Bilateral GODIVA

**FIGURE 5:**
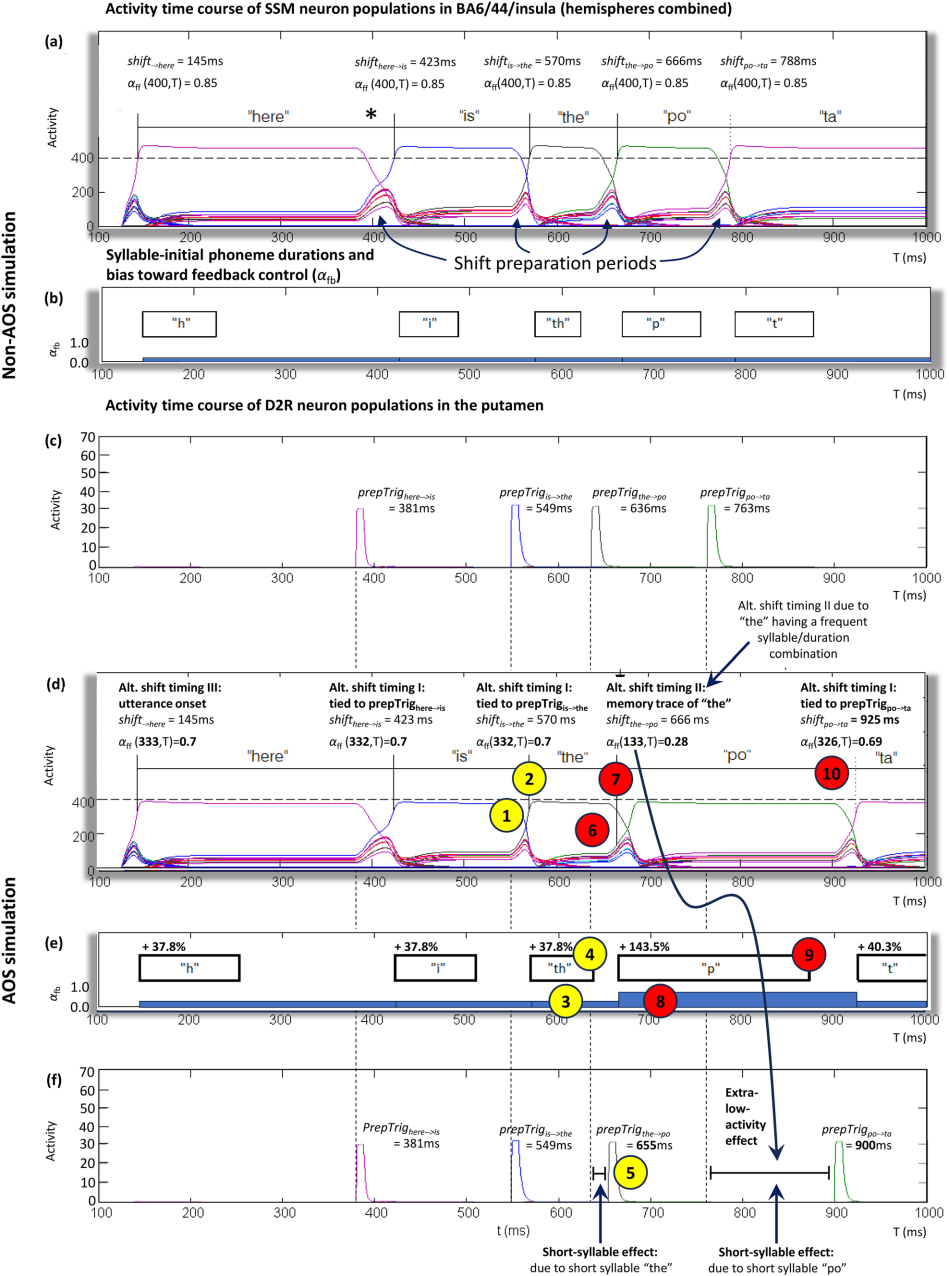
Time courses of neural activity and speech output in non‐AOS (a–c) and AOS (d–f) simulations. Circled numbers in the AOS simulation panels indicate the point of each numbered step from Figure 4, and text in bold indicates differences from the non‐AOS simulation. The utterance produced by the model is ‘here is the potato’ (the periods of the syllables are given in panels a and d). The activity of neuron populations corresponding to different syllables is plotted in distinct colours, with the colours of ‘here’ and ‘is’ matching those in Figure 1. The ranges of activity levels (*y*‐axes) have arbitrary units: the units in panels (c, f) were inherited from Civier et al. (2013) as‐is, and those in panels (a, d), scaled down by 100 for readability. (a, d) SSM neuron populations, summed activity across the two hemispheres. A horizontal dashed line indicates the normal‐readout threshold, which is set at 80% (400) of the maximum possible BA6/44/insula activity (500). Full vertical lines indicate syllable shifting points, and report the shift‐timing method, the time of the shift, and the formula used to calculate the feedforward control weighting (see Equation 1). (b, e) For each syllable, duration of syllable‐initial phone (upper rectangles) and bias towards feedback control (bottom bars). Panel (e) also indicates the percentage prolongation of each phone in AOS. (c, f) Putamen D2R neuron populations, whose activity is inhibitory to the SSM neuron populations (see Supporting Information). The timing of each shift‐preparation trigger is indicated, and dashed vertical lines go down from panel (c) to (f) to allow comparison between triggers in the non‐AOS and AOS simulations. Additional regions in the model (Figure 1) are not presented for simplicity: IFG functionality is normal in AOS, GPe, GPi and thalamus are relaying the putamen output signals (see Supporting Information), and neural dynamics in vMC are not explicitly modelled here (Section 3.2.3). SSM = Speech Sound Map. D2R = D2 dopamine receptors. Alt. shift‐timing = Alternative shift‐timing mechanism which is *not* based on SSM neuron population activities reaching the normal‐readout threshold, i.e., dashed line (see Section 3.2.4 for details on the different alternatives). *shift*_*x➔y*_ = shift from syllables x to y. *prepTrig*_*x➔y*_ = the trigger to prepare for shifting from syllables x to y.

The simulations assume that all the syllables in the utterance ‘here is the potato’ are part of the syllabary and have SSM populations coding for them (see Bohland et al. 2010). The syllable durations—which include both the voiced and unvoiced parts of each syllable—were taken from a representative neurotypical speaker producing the same utterance (one of the participants we studied in Vergis et al. 2014): 278 ms for ‘here,’ 147 ms for ‘is,’ 96 ms for ‘the’ and 120 ms for ‘po’ in ‘potato.’ As speech output in the non‐AOS simulation is normal, these are the exact durations displayed in Figure 5a. Notice that for the sake of the simulation, only ‘the’ and ‘po’ will be considered short syllables. The durations of the initial phones within each syllable were based on typical values suggested by Kent and Read (1992). The only exception was the /ɪ/ in ‘is.’ We set its duration to half of the suggested value of 130 ms to account for ‘is’ being a frequently occurring unstressed function word with shorter vowel duration (Umeda 1975). To run the simulations, we used a MATLAB (http://www.mathworks.com/) implementation of the bilateral GODIVA model, numerically integrating the equations using a time step of 0.33 ms.

#### 3.2.6 Comparison With Published Acoustic Measurements

Comparing the simulation results with experimental data from cases with pure AOS is challenging due to its rarity (also see Section 2.2.1). Instead, we will compare the mean syllable lengthening percentage between AOS and non‐AOS simulations to acoustic data from Vergis et al. (2014) for AOS‐plus‐aphasia and aphasia‐only participant groups. That study reported that the average initial syllable duration for the polysyllabic word terminating ‘here is the ____’ was 94 ms for the AOS‐plus‐aphasia group and 51 ms for the aphasia‐only group (*p* = 0.003, Vergis et al. 2014, durations extracted from Figure 3 of the same paper). These two values were averages for multiple unstressed‐stressed target words, including the word ‘potato’ used here. This allows us to calculate a single syllable lengthening value (88%) to compare simulation results.

Note that the participants from Vergis et al. (2014) and those from the neuroimaging experiment were drawn from the cohort reported in Ballard et al. (2016). For adults with AOS‐plus‐aphasia, 6 of the 9 participants from Vergis et al. and the 15 from the neuroimaging experiment overlapped. For adults with aphasia‐only, 5 of the 8 and 11 participants, respectively, overlapped. The participant description in Section 2.2.1 applies to them all (see also Table 2).

### 3.3 Results

#### 3.3.1 Non‐AOS Simulation

Figure 5a–c shows the output of a non‐lesioned version of the bilateral GODIVA model producing ‘here is the potato,’ hence the non‐AOS simulation.

According to Figure 5a, each of the SSM neuron populations that initiated a syllable, was selected by surpassing the normal‐readout threshold. As such, motor programs were read out normally, with the baseline *α*_ff_ value of 0.85, and the timing of syllable shifts was standard, that is, syllables initiated as soon as the populations' activities reached threshold. Rather than describing syllable production one by one, below we will only concentrate on the first shift between syllables (‘here’ to ‘is,’ also depicted in Figure 1), as it gives a good representation of the process. The special cases of initiating and terminating the utterance are less relevant to the current investigation and will not be discussed here (for more details, see Civier et al. 2013).

The process of shifting from ‘here’ to ‘is’ begins with a shift preparation period from *t* = 381 ms and on. Driven by the detection of the imminent completion of the ‘here’ syllable, the syllable's putamen D2R neuron population issues a shift‐preparation trigger (transient strong activity in Figure 5c, magenta). This D2R neuron activity is inhibitory to cortex and, shortly after, it quenches the activity of the SSM neuron population for ‘here’ (Figure 5a, magenta as well). In the meantime, the SSM neuron population for ‘is’ is the best match for the phonological units of the next syllable (/ɪ/ followed by /z/), and as such, it receives strong upstream input from both the 4th‐position /ɪ/ and 5th‐position /z/ neuron populations in the inferior frontal gyrus, or IFG (see Figure 1) (Bohland et al. 2010; Civier et al. 2013). Indeed, for hundreds of milliseconds already, the population for ‘is’ had the highest activity among the syllables competing to succeed ‘here’ (Figure 5a). Now that the SSM population for ‘here’ is being quenched, the ‘is’ population increases its activity to become dominant in BA6/44/insula, and starting at t = 410 ms, it exerts enough lateral inhibition to shut down the other competing neuron populations. At *t* = 423 ms it reaches the normal‐readout threshold and initiates the syllable ‘is’. The shifting between the rest of the syllables in the utterance is conducted in a similar fashion.

#### 3.3.2 AOS Simulation

Figure 5d–f show the same data for the AOS simulation, where the model has 17.8% of the SSM neurons eliminated. As evident from Figure 5e, the initial phones of all syllables are prolonged by at least 37.8%, with a maximum prolongation of 143.5% occurring on the phone ‘p’ of ‘po.’ Nevertheless, strictly in terms of syllable durations (Figure 5d), there is no difference compared to the non‐AOS simulation for most of the syllables in the utterance (including ‘ta’ and ‘to,’ not shown here). The sole difference in duration occurs in the syllable ‘po,’ which is much longer in the AOS simulation (compare the distance between *shift*_*the➔po*_ and *shift*_*po➔ta*_ in Figure 5a,d).

#### 3.3.3 Match to Acoustic Data

As hypothesised, the AOS simulation produced the word ‘potato’ abnormally. The duration of the syllable ‘po’ in ‘potato’ was 259 ms in the AOS simulation, compared with only 122 ms in the non‐AOS one. This accounts for AOS‐related lengthening of 112% and is qualitatively similar to the group‐averaged lengthening of 88% calculated from Vergis et al. (2014) (Section 3.2.6). In both cases, the abnormally produced syllable is about double in length. The duration of ‘ta’ in ‘potato’ was not different between the non‐AOS and AOS simulations (0% lengthening), consistent with the published acoustic data.

Although lengthening extent in the simulation agrees with published experimental data, actual syllable durations were markedly different. In the non‐AOS simulation, for example, word‐initial syllable duration was 122 ms but 51 ms in Vergis et al. (2014). This is due to the simulation dealing with total syllable duration, whereas Vergis et al. only considered the voiced portion of the syllable. As the voiced portion is proportional to total syllable duration, both measures should be equally affected by lengthening. Another factor contributing to the difference is that Vergis et al. averaged measurements across several words with the unstressed–stressed pattern, whereas the simulation used only the word ‘potato.’

### 3.4 Discussion

The goal of this modelling experiment was to propose and test the plausibility of a mechanistic explanation for a lexical stress alteration characteristic of AOS; namely, lengthening of word‐initial unstressed syllables (e.g., ‘po’ in potato). We implemented the mechanistic explanation within the framework of the neuro‐computational GODIVA model, ‘lesioning’ the model's BA6/44 region, consistent with the site of lesion in stroke‐induced AOS. We then simulated production of the sentence ‘here is the potato’ and examined speech output abnormalities in ‘potato.’

The AOS simulation indeed shows abnormal speech output in ‘potato’: the phone ‘p’ is prolonged, and the syllable ‘po’ as a whole is lengthened. Moreover, the syllable lengthening in the simulation resembles a statistically significant lengthening reported in patients with AOS (Vergis et al. 2014). The agreement of the current simulation with previously published acoustic data supports the plausibility of our mechanistic explanation; namely, that syllable lengthening in AOS (in this case, ‘po’) is due to time‐varying bias towards feedback control, which was exacerbated during the execution of the preceding syllable (in this case, ‘the’).

Below we will discuss the altered neural dynamics that gave rise to the atypical speech output in the AOS simulation. As shown in Figure 5d–f (info in bold), the neural dynamics in the AOS simulation differed from the non‐AOS simulation in several ways, beginning well before the syllable ‘po.’ Therefore, we will take a wider‐lens approach and examine neural dynamics all along the utterance.

#### 3.4.1 Global Effect: Prolongation of Syllable‐Initial Phones

As we predicted, the ‘lesion’ in the model resulted in no SSM neuron populations reaching threshold for syllable readout (Figure 5d), with activity never surpassing 390 (i.e., 97.5% of the required threshold of 400). Nevertheless, shifts between syllables still took place, driven by the alternative shift timing mechanisms that can force readouts when a population's activity is below threshold (Section 3.2.4). The drawback is that motor program readout will be weaker, that is, *α*_ff_ < 0.85, with the exact value of *α*_ff_ being relative to SSM neuron population activity at time of syllable shifting. For example, the SSM neuron population for ‘the’ has an activity level of 332 at the time of shift (*t* = 570 ms), and thus, the syllable ‘the’ is produced with *α*_ff_ = 0.70 (according to Equation 1, $\alpha_{\mathrm{ff}}=0.85\times \frac{s\left(t\right)}{T}=0.85\times \frac{332}{400}=0.70$). The reduced *α*_ff_ results in abnormal articulation of all syllables due to reliance on feedback control (see Section 1), and Figure 5e shows that prolongation of the initial phone indeed occurs in all of them. Continuing with the example of ‘the,’ using $\%\text{chang}e_{\alpha_{\mathrm{ff}}}^{"\mathrm{th}"}$ = −18% in Equation (3) (*α*_ff_ = 0.7 is 18% smaller than 0.85) results in the initial phone of the syllable being prolonged by 37.8%. These initial phone prolongations are in agreement with findings from the literature indicating prolonged consonant duration in AOS (Bauman 1978; Kent and Rosenbek 1983; Seddoh et al. 1996).

#### 3.4.2 Short‐Syllable Effect: Delays *prepTrig_the➔po_* and *prepTrig_po➔ta_*

In the AOS simulation, the prolongation of the initial phone of each syllable also delays the syllable's subsequent phones, including its final phone (Figure 5e). The final phone of each syllable includes an articulatory configuration that cues a shift‐preparation trigger (see Supporting Information), and this cueing is logically delayed as well. For example, due to a 37.8% prolongation of ‘th’ in ‘the,’ the syllable's final phone ‘e’ starts late (Figure 5e), which translates to postponed cueing of *prepTrig*_*the➔po*_ (Figure 5f). It is cued at *t* = 655 ms, compared with *t* = 636 ms in the non‐AOS simulation (Figure 5c).

The impact of initial phone prolongation on the remainder of the syllable appears to be dependent on context—in all short syllables (short monosyllabic words or unstressed syllables), the cue is delayed like in the case of ‘the’ (‘short‐syllable effect’ in Figure 5f). However, in long syllables, the cueing can be moved earlier towards the beginning of the final phone (or even the end of the syllables' nucleus), thus preventing a delay. For example, in the AOS simulation, the ‘i’ in ‘is’ is prolonged by 37.8% which delays the onset of ‘s’ (Figure 5e). However, instead of *prepTrig*_*is➔the*_ being delayed as well, it is moved to an earlier position in ‘s,’ successfully preserving the cue original time of 549 ms (Figure 5f) and implying a ‘catching up’ effect. The exact method by which the shift‐preparation trigger is moved earlier in long syllables is not the focus of this work and has been implemented algorithmically. For more biologically realistic simulations, it can be modelled by increasing the sensitivity of the shift‐trigger cueing mechanism in GODIVA (see Supporting Information).

The ability of long syllables to ‘catch‐up’ with the original cueing time, hence evading the short‐syllable effect, is owing to their phones being long and to having the shift‐preparation trigger well into the final phone, like *prepTrig*_*is➔the*_ in Figure 5c (compare with short syllables which have short final phone; the trigger must be positioned early in the phone to begin with, see *prepTrig*_*the➔po*_ in Figure 5c, and there is no room for compensation, Figure 5f). Moreover, it predicts that the final phone of long syllables will be truncated to compensate for the prolongation in the initial phone. This is indeed the case—whereby ‘s’ in the AOS simulation was shorter than in the non‐AOS simulation (compare Figure 5b and Figure 5e).

#### 3.4.3 Extra‐Low‐Activity Effect: Delays *prepTrig_po➔ta_* Further

The shift‐preparation triggers are delayed when preparing to shift out of both ‘the’ and ‘po,’ but it is evident from Figure 5f that the delay in *prepTrig*_*the➔po*_ is smaller compared with *prepTrig*_*po➔ta*_. This can be sourced all the way to the activity of the corresponding SSM neuron populations when ‘the’ and ‘po’ are initiated. Note that SSM activity of ‘the’ at initiation is higher than that for ‘po’ at initiation (332 vs. 133, Figure 5d) and the delay in preparing to shift out of ‘the’ is consequently smaller (655 − 636 = 19 ms delay for *prepTrig*_*the➔po*_ vs. 900 − 763 = 137 ms delay for *prepTrig*_*po➔ta*_, Figure 5c,f; also see horizontal bars in Figure 5f).

The effect of the activity level on the delay in shift‐preparation trigger is achieved through a chain of causal interactions: (1) the activity level decides *α*_ff_ (Equation 1), (2) *α*_ff_ decides the prolongation of the initial phone of the syllable (Equation 3), and (3) the prolongation of the initial phone decides the delay in the onset of the final phone, (4) the onset of the final phone decides the delay in the shift‐preparation trigger. Briefly, 1, 3, and 4 are direct relationships, but because 2 is an inverse relationship (i.e., lower weighting for reading the motor program results in prolonged movements, and the compensation by feedback control is slow as well; see Section 1), the effect as a whole is inversed, that is, lower activity level leading to greater delay.

For the case of ‘po,’ the above chain of interactions is as follows: (1) activity level of 133 gives *α*_ff_ = 0.28 (Figure 5d), (2) *α*_ff_ = 0.28 stands for $\%\text{chang}e_{\alpha_{\mathrm{ff}}}^{"p"}$ = −70%, and thus, 143% prolongation of the initial phone ‘p’ (Figure 5e), (3) the onset of ‘o’ is substantially delayed (Figure 5e), and (4) *prepTrig*_*po➔ta*_ is delayed by 137 ms (Figure 5f). Thus, although SSM activity is always sub‐threshold in the AOS simulation, the extreme low activity when initiating ‘po’ leads to a larger impact on the shift to the next syllable.

#### 3.4.4 Extremely Delayed *prepTrig_po➔ta_* Leads to Extreme Lengthening of ‘po’

Like most syllables in the AOS simulation, the speech system times the shift from ‘po’ to ‘ta’ using alternative shift‐timing I, which is tied to the corresponding shift‐preparation trigger, *trigPrep*_*po➔ta*_. As *trigPrep*_*po➔ta*_ experiences a large delay (Figure 5f), so does the shifting into ‘ta’ (*shift*_*po➔ta*_ in Figure 5d). The delayed shifting into ‘ta’ means that ‘po’ keeps executing for longer, and thus is being lengthened.

In contrast with the lengthening of the syllable ‘po’ of ‘potato,’ the next syllable of the word, ‘ta,’ has a normal duration (not shown in Figure 5 due to space constraints). ‘ta’ being a long syllable, is not affected by the Short‐syllable effect and, being initiated by the SSM neuron population with only moderately low activity (Figure 5d), is also not affected by the Extra‐low‐activity effect. Given that the word‐initial short syllable ‘po’ is lengthened whereas the second‐position long syllable ‘ta’ is not, the difference between their durations diminishes. This results in a reduced stress contrastiveness within the word ‘potato’ in the AOS simulation. It is also consistent with acoustic data from Vergis et al. (2014) which reports that individuals with AOS show lower values on the pairwise variability index (PVI), a measure of lexical stress contrast, on polysyllabic words with unstressed initial syllables (also see Ballard et al. 2016; Ballard et al. 2014; Landin‐Romero et al. 2021).

#### 3.4.5 Conditions for Extra‐Low‐Activity Effect: Preceding Short Syllable That Has a Frequent Syllable/Duration Combination

The direct cause for the lengthening of ‘po’ is the delay in the shift‐preparation trigger, *prepTrig*_*po➔ta*_. However, the delay and lengthening were exacerbated by the Extra‐low‐activity effect. As AOS is characterised by extreme lengthening of syllables, it is warranted to understand when syllables are initiated with extremely low activity, as is the case of ‘po.’

Analysing the neural dynamics of the AOS simulation, it is evident that the extremely low activity of the ‘po’ syllable's SSM neuron population is an outcome of two factors. Both are associated with the properties of the prior syllable ‘the.’ First, ‘the’ is short and the Short‐syllable effect delays the shift‐preparation trigger *prepTrig*_*the*➔*po*_ (Figure 5d); second, ‘the’ has a frequent syllable/duration combination and, thus, the shifting from ‘the’ to ‘po’ is timed using Alternative shift‐timing II (Section 3.2.4). Recall that according to this shift‐timing method, the shift‐preparation trigger is disregarded, and instead, the well‐established memory trace of the duration of ‘the’ (96 ms, see Section 3.2.5) is used to time the shift.

What happens then when the trigger to prepare the shift from ‘the’ to ‘po’ is delayed, whereas the shift itself takes place at the memorised time, hence is not delayed? Because *prepTrig*_*the➔po*_ is late (Figure 5f), the preparation process that involves activating the SSM neuron population for ‘po’ is obviously delayed as well. This contrasts with the normally timed shift, leading to a situation where the SSM neuron population for ‘po’ does not have sufficient time to prepare prior to the shift (from *prepTrig*_*the➔po*_ to *shift*_*the➔po*_ is just 11 ms; Figure 5d,f). Indeed, the population for ‘po’ fails to increase its activity in time for *shift*_*the➔po*_ (Figure 5d, green time course; 133 is only 33% of the threshold activity level), explaining why the Extra‐low‐activity effect applies to the production of the syllable ‘po’ in the simulation.

#### 3.4.6 Extreme Lengthening of ‘po’ When Preceded by ‘the’: Summary and Generalisation

Above we described several effects that interact to produce an extreme syllable lengthening at the specific context of ‘the potato.’ First, the syllable ‘po’ is short, and thus, *prepTrig*_*po➔ta*_ is delayed (Short‐syllable effect). Second, the preceding syllable ‘the’ is both short and a frequent syllable/duration combination, leading to ‘po’ being initiated with extremely low activity (Extra‐low‐activity effect) that further delays *prepTrig*_*po➔ta*_. The extreme delay in starting the preparation for shifting from ‘po’ to ‘ta’ produces an extremely late shift, which effectively lengthens ‘po.’ The second effect (Extra‐low‐activity), in particular, blames the preceding syllable ‘the’ for the lengthening of ‘po,’ hence aligning with our mechanistic explanation.

Taking our findings from simulation to the real world, the neural dynamics in the simulation imply that any syllable with comparable properties to ‘po’ in potato would be equally lengthened when preceded by ‘the.’ Support for that contention arrives from the Vergis et al. (2014) study that we used to evaluate the simulation. The lengthening reported by Vergis et al. is actually the average lengthening in a set of word‐initial syllables similar to ‘po’ (i.e., unstressed short syllables), suggesting that the culprit is not the specific syllable ‘po’ but rather its short duration. In line with our mechanistic explanation, we suggest a general pattern whereby when a short syllable with a frequent syllable/duration combination (e.g., ‘the’ or ‘an’) is followed by another short syllable (e.g., ‘po’ in potato or ‘e’ in echidna), the latter will be substantially lengthened. On the other hand, we predict that other utterances will be spared from the extreme lengthening, as they do not fully match the criteria. In ‘There is no potato’, for example, we predict that ‘po’ will not be substantially lengthened as the preceding monosyllabic word ‘no’ is not short.

## 4 General Discussion and Conclusions

We presented and empirically supported the plausibility of a novel mechanistic explanation for the alteration in lexical stress characteristics of acquired AOS (see Figure 4). Briefly, a lesion affecting the SSM (i.e., the motor program store) leads to neural populations for syllables to be always under‐activated. This biases the speech production system away from feedforward control and towards feedback control (see Section 3.2.3), prolonging all syllable‐initial phones. In some cases, this results in a subsequent short syllable not having sufficient preparatory neural activity (e.g., ‘po’ is triggered with an activity level of only 133 in Figure 5d; see Extra‐low‐activity effect in Section 3.4.5), and so even greater reliance on feedback and substantial lengthening of the short syllable (see Sections 3.4.3 and 3.4.4 for details). Because the effect is context‐dependent, typically only one syllable—an unstressed syllable—is extremely lengthened in each word. The lengthening reduces the contrast between the unstressed syllable and the adjacent stressed syllable, thereby altering the word's lexical stress pattern.

The above mechanistic explanation was partly guided by a neuroimaging experiment. The neuroimaging experiment analysed structural and functional MRI in a sample of participants with and without AOS, and suggested that: (1) on average, the lesion in AOS destroys 22.25% of the *left‐hemisphere* neurons of each syllable's motor program, or 17.8% total destroyed neurons, (2) the connectivity from the left lateral SSM to the right motor cortex is reduced in AOS (expanded to both lateral and medial SSM, and to both left and right motor cortices based on statistical and theoretical considerations, see Section 2.4.2), and (3) the activities of left and right motor cortices are less synchronised in AOS, which may indicate that instead of both being controlled identically by feedforward motor control, the right motor cortex is also being overly influenced by the intact feedback motor control network in the right hemisphere.

The mechanistic explanation's plausibility was then tested by an AOS simulation of the bilateral GODIVA model (see Section 3.2 for the changes introduced to the model). It showed that: (1) the hypothesised lesion in AOS leads to extreme lengthening of word‐initial short (unstressed) syllables, and particularly, when the word is preceded by another short syllable of certain characteristics (frequent syllable/duration combination, see Section 3.4.6), (2) the amount of lengthening in the model is comparable to that observed in a previous acoustic study of AOS that used the same linguistic constructs, and (3) although the lengthening was demonstrated in a single exemplar utterance, the neural dynamics should generalise to other utterances with similar properties. Most importantly, point 1 above allowed us to refine our mechanistic explanation to certain linguistic contexts, which warrants testing in future studies.

Our explanation places emphasis on the role of the syllable that precedes the unstressed syllable being lengthened in AOS (e.g., ‘the’ that precedes ‘po’ in the simulated sentence). Specifically, we conclude that the preceding syllable, if short and having a frequent syllable/duration combination, would magnify the lengthening. We did not directly simulate a case where a preceding syllable does not have these properties, but the literature provides a relevant test case (Vergis et al. 2014). Vergis et al. compared individuals with AOS‐plus‐aphasia and aphasia‐only, using stressed–unstressed words (e.g., *dinosaur*) embedded in sentences. In these words, the unstressed syllable (‘no’) is preceded by a stressed, hence, long syllable (‘di’). Although there was still lengthening of the unstressed syllable of the words (*p* = 0.039), it was less pronounced than the lengthening that occurred in the same study when the unstressed syllable is word‐initial, and preceded by a short syllable such as ‘the’ (*p* = 0.003, see Section 3.2.6). This aligns well with our contention that without a preceding syllable with the specific characteristics that we outline, the unstressed syllable's lengthening in AOS—and subsequently, the lexical stress alteration—are indeed less pronounced. We recommend that future studies will more precisely quantify the relative contributions of each contextual effect to perceived dysprosody (preceding syllable being short vs. preceding syllable having a frequent syllable/duration combination). Taking these context‐dependent effects into account may improve stimulus design for both diagnostic assessment and behavioural intervention.

As evident from the simulations and the previous paragraph, the model presented here is focused primarily on syllable duration. However, syllable‐initial phone duration is simulated and discussed as well (see Figure 5e and Section 3.4.2). Specifically, the model predicts longer initial phone durations (Figure 5e), which aligns well with experimental data. Previous studies found that patients with AOS tend to have longer syllable‐initial phone durations (Bauman 1978; Kent and Rosenbek 1983) and show significantly longer stop gap and consonant‐vowel (CV) durations (Seddoh et al. 1996). These studies also report the phone prolongation pattern to be highly variable, which again agrees with the contextual effects observed in our simulation (different prolongation ratios for the syllable‐initial phones in Figure 5e). Because the model does not explicitly simulate the durations of non‐initial phones (other than initial phones, we only simulate total syllable durations, see Section 3.2.4), it cannot make specific predictions on alterations of these late‐syllable segments in AOS. That said, being the vocalic/post‐vocalic portion of the syllable, we expect their durations to be affected by the assumed reliance on feedback control (Tilsen 2022). We recommend that future behavioural studies of AOS provide a more complete picture of both initial and non‐initial phone durations to allow testing of distinct theoretical models: equal versus differential phone lengthening, lengthening proportional to phone duration versus absolute, and so forth (e.g., Tilsen 2022).

The instantiation of the DIVA/GODIVA model used here makes assumptions about the functional specialisation of the left and right hemispheres at different motor processing stages. At the level of premotor cortex, feedforward control is mostly left lateralised (80% of left SSM neurons and only 20% on the right), but a hypothetic complete destruction of the right‐hemisphere SSM neurons would still result in neuron loss that is comparable to the 17.8% loss simulated here. This agrees with the fact that AOS in left‐dominant individuals with right‐hemisphere lesion exists but is fairly rare (Balasubramanian and Max 2004). At the level of motor cortex, in contrast, feedforward control is not lateralised (i.e., 50% left‐ and 50% right‐hemisphere neurons), suggesting that left and right motor cortices should have synchronised activities across time; indeed, we observed high functional connectivity between the motor cortices in non‐AOS individuals.

The above picture is quite different for feedback control, which is right lateralised at all processing levels. This allowed us to interpret the reduced functional connectivity between the motor cortices in AOS as an indication that the unique motor control capabilities of the right motor cortex (i.e., feedback control) are suddenly engaged. The above assumptions about lateralisation in premotor and motor cortices were popularised by the DIVA model (Golfinopoulos et al. 2010; Guenther 2016), and we hope that their utility in explaining aspects of AOS would contribute to their wider acceptance in the research community.

One strength of this study is the use of multiple modalities: structural and functional MRI (analysed here) as well as acoustics (taken from a previous publication). Another strength is that the participants that provided MRI data and those that provided acoustics data were all part of a single cohort (Ballard et al. 2016), hence, recruited using the same criteria. Ideally, we would limit the analysis to only those participants that provided both MRI and acoustics data, but unfortunately, the resulting limited subset (5 aphasia‐only participants, 6 AOS‐plus‐aphasia participants) was too small for statistical analysis.

It is important to emphasise that, in this study, the model only provides an explanation of already available behavioural and imaging data from AOS patients. However, future experiments should prospectively model predictions from our simulations for behaviour and functional connectivity. For example, the contextual effects (i.e., sequences of long vs. short syllables with high vs. low frequency) on speech acoustics in AOS can be tested with carefully designed stimulus sets. Also, neuromodulation, neurofeedback or possibly pharmaceuticals that can alter the underlying neural deficiency of abnormally low SSM activity might prove fruitful. We foresee neuro‐computational models, such as the one used here, to be instructive in the development of such interventions, whether for fine‐tuning of stimulation parameters, designing efficacy experiments or predicting possible side effects.

Future experiments should also focus on a major assumption we took in this study: that a degeneration in the connections from the left SSM medial and lateral to the left Articulator map existed, but due to technical limitations, could not be detected using our functional connectivity analysis (Section 2.4.2). One approach to detect the assumed impairment might be to investigate the structural rather than the functional connectivity between these regions. Another option involves using a more sensitive functional connectivity method, such as voxel‐based analysis or one with subject‐specific ROI localisation. If evidence consistently argues against the degeneration of left‐hemisphere connections, our model would have to be revised; for example, by assuming left‐hemisphere plasticity that re‐establishes SSM‐to‐motor connectivity, possibly via an alternative pool of neurons.

This study has some limitations related to the participant sample used. The participants that contributed MRI data were from a consecutive sample recruited to a larger study and represented those who passed screening for and consented to MRI scanning; therefore, the sex ratio was not controlled. This resulted in several females being included in the aphasia‐only and neurotypical groups compared with no females in the AOS‐plus‐aphasia group. The current participant sample is too small to evaluate the effect (if any) of the uneven sex ratio between the groups, but further examination into this possible confound is warranted.

The design of the model and the extent of the simulated lesion were based on our specific cohort of AOS. Findings from this study may have limited generalisability to individuals with more severe speech or language impairment. That said, the fact that altered lexical stress is such a powerful predictor of AOS in English suggests that the abnormality simulated here is a common feature of AOS. Because our cohort consisted solely of Australian English speakers, generalisability across languages might be limited as well. That said, the initial unstressed syllable lengthening effect for words such as ‘potato’ has been demonstrated in both Australian and American English, which suggests a more general pattern for stress‐timed languages. Indeed, the importance of lexical stress in facilitating speech production in AOS has been demonstrated in another stress‐timed language, German, where speakers show more speech errors in words with unstressed‐stressed patterns (e.g., Aichert et al. 2016; Ziegler and Aichert 2015) and difficulty controlling syllable durations for stress‐timing (Brendel and Ziegler 2008). We encourage additional work on how the mechanism described here could manifest acoustically for other stress‐timed languages including underrepresented ones. Furthermore, given that lexical stress is a symptom of an underlying motor planning disorder, there is already increasing work exploring how to measure this temporal disruption in languages that are not stress‐timed, such as Spanish and Cantonese (Wong et al. 2024).

Other limitations concern the ROI selection. In this study we used fixed anatomical locations for our SSM ROI as defined in the DIVA model, which are in turn based on results of past neuroimaging studies (Guenther 2016). These coordinates are meant to represent the ‘population average’; that is, they do not account for individual variability in the exact location of the SSM. A more sophisticated approach using individual‐specific ROIs identified using functional localiser scans (Fedorenko et al. 2010; Nieto‐Castanon et al. 2003; Nieto‐Castañón and Fedorenko 2012) may be warranted in future studies to account for inter‐individual variability in the location of functional areas and to avoid cases where a single ROI corresponds to different functional regions across individuals (see Fedorenko and Blank 2020).

Finally, when designing the simulations, we had to take assumptions (e.g., reduced functional connectivity may indicate reduced synchronisation rather than structural deficiency, see Section 2.4.3) and make compromises (e.g., due to the difficulty in imaging subcortical structures, the imaging experiment could not guide modelling for that part of the brain, see Section 2.1). This is an inevitable part of computational modelling, where experimental evidence is often limited in scope and details compared to the model itself. With improvements in experimental methods, the model will be progressively updated and refined to reflect newly available data.

## Ethics Statement

The study was approved by the Sydney Southwest Area Health Service and the University of Sydney Human Research Ethics Committees, with all procedures adhering to the ethical principles of the Declaration of Helsinki.

## Consent

All participants gave their informed written consent prior to their inclusion in the study.

## Supporting information


**Data S1:** hbm70412‐sup‐0001‐supinfo.docx.

**Data S2:** hbm70412‐sup‐0002‐supinfo.docx.

## Data Availability

The data that support the findings of this study are available on request from the corresponding author. The data are not publicly available due to privacy or ethical restrictions.
