# Correction to “An Imaging‐Guided Neural Model Explains Lexical Stress Alteration in Acquired Apraxia of Speech”

**DOI:** 10.1002/hbm.70487

**Published:** 2026-02-23

Civier, O. , A. Ramage , J. Tourville , D. A. Robin , F. H. Guenther , and K. J. Ballard . 2025. “An Imaging‐Guided Neural Model Explains Lexical Stress Alteration in Acquired Apraxia of Speech.” Human Brain Mapping 46, no. 17: e70412. 10.1002/hbm.70412.41378706 PMC12696586

In the original online publication, funding information from the National Health and Medical Research Council grant 2037102 was missing.

The corrected text is as follows:

**Funding:** The work was funded by an Australian Research Council Future Fellowship FT120100355 and National Health and Medical Research Council grant 2037102 to Ballard, and a National Institutes of Health/National Institute on Deafness and Other Communication Disorders grant R01DC007683 to Guenther and Tourville.

**Acknowledgements**

The work was funded by an Australian Research Council Future Fellowship FT120100355 and National Health and Medical Research Council grant 2037102 to Ballard, and a National Institutes of Health/National Institute on Deafness and Other Communication Disorders grant R01DC007683 to Guenther and Tourville. The authors acknowledge the scientific and technical assistance of the National Imaging Facility, a National Collaborative Research Infrastructure Strategy (NCRIS) capability, at Swinburne Neuroimaging, Swinburne University of Technology. We are grateful to the Department of Cognitive Science, Macquarie University, and especially to Prof. Paul Sowman, for facilitating the travel of Civier to Australia.

We apologize for this error.

