# Supplementary material for: An Imaging‐Guided Neural Model Explains Lexical Stress Alteration in Acquired Apraxia of Speech
Source: Hum Brain Mapp. 2025 Dec 11;46(17):e70412. doi: 10.1002/hbm.70412 (PMC12696586; doi:10.1002/hbm.70412)
Supplement: Supplementary file 1 — Data S1: hbm70412‐sup‐0001‐supinfo.docx. [file HBM-46-e70412-s001.docx]

**Supporting Information for**

An imaging-guided neural model explains lexical stress

alteration in acquired apraxia of speech

Oren Civier, Amy Ramage, Jason Tourville, Donald A. Robin, Frank H. Guenther,
Kirrie J. Ballard

# The bilateral GODIVA model for neurotypical adults

For the computational simulations, we used the bilateral GODIVA model. It is a modification of the extended GODIVA model (Civier et al., 2013), which itself, is an extension of the original Gradient Order DIVA (or GODIVA) model (Bohland et al., 2010). The bilateral GODIVA model explains how arbitrary utterances that fall within a speaker’s language rules can be represented in the brain, and how such utterances can be produced from a finite library of learned motor programs when activated in the proper order. Each motor program stands for a well-learned syllable, and as such, the model disregards words, and simply treats an utterance as a sequence of syllables. To imitate the process of speech production, the model simulates the activity of multiple neuron populations in the brain, and the interactions between them.

The bilateral GODIVA model is implemented computationally as a neural network governed by a set of ordinary differential equations that capture key features of membrane dynamics, including the bounded range of cell potentials (Grossberg, 1978). However, being a high-level cognitive model, the bilateral GODIVA model does not simulate the firing of each neuron in the system, but rather groups neurons into neuron populations. Each population is a single computational unit, and at each moment in time t, it has an activity in a certain range (e.g., 0 to 500 in arbitrary units). The activity of a neuron population is decided by the activities of populations projecting to it, the types (excitatory or inhibitory) and strengths of the projections, and the population’s spontaneous decay (or recovery) rate. Moreover, the activity of a neuron population is also regarded as the output of the population, and the same output signal is transmitted to all efferent projections (Grossberg, 1978). Fig. 1 in the main text shows the regions that constitute the bilateral GODIVA model, and the connections between them. For the cortical regions, only the layer where neuron populations are actually being activated ("choice" layer) is shown and discussed. The layer where neuron populations are primed for selection ("planning" layer) is omitted for simplicity and because it does not play a role in AOS. More information on the roles of layers in this type of models is available elsewhere (Bohland et al., 2010; Brown et al., 2004).

## Feedforward control system: Bilateral Speech Sound Map and Articulatory Map regions

This study focuses on the neuron populations in the Speech Sound Map (SSM), assumed to be implicated in AOS, and roughly corresponds to BA6/44 (ventral premotor cortex/posterior inferior frontal gyrus) on the left hemisphere (Golfinopoulos et al., 2010), and the anterior insula on both left and right hemispheres (Guenther, 2016). The function of the SSM neuron populations is similar to the “Mental syllabary” mentioned in the intro (Levelt et al., 1999), with each population encoding a motor program for a distinct well-learned syllable (Fig. 1 in the main text). This part of the bilateral GODIVA model is labelled ‘feedforward control system’ in reference to the automated nature of the motor programs (Guenther et al., 2006).

In order to produce a syllable, the brain needs to select and rump up the activity of the corresponding SSM neuron population (shown in Fig. 1 in the main text is the process of increasing the activity of the “is” syllable). Once a SSM neuron population reaches the normal-readout threshold (dashed horizontal line in Fig. 5a in the main text; the figure presents a simulation of a neurotypical bilateral GODIVA model), it starts reading out the motor program for the corresponding syllable (or in short, initiates the syllable). The normal-readout threshold is set to activity level of 400 – a point where a neuron population indeed has enough activity to read out its motor program in a normal, intact fashion. As the activity range of SSM neuron populations (0-500) has arbitrary units that are model-inherent, we will often report SSM neuron activity as a percentage of the normal-readout threshold level (i.e. 0-125%).

The readout of the next syllable motor program also terminates the previous syllable, thus, in effect, shifts between previous and next syllables (Fig. 5a in the main text, vertical lines). Each motor program is sent to the Articulator Maps (bilateral motor cortices) for motor execution. Actual syllable execution was modelled previously by the DIVA model (Guenther et al., 2006), but because we assume an intact motor cortex in AOS, this part of the speech production system will not be simulated here. Instead, we will algorithmically estimate the expected duration of segments within each syllable (see phone periods in Fig. 5b in the main text).

Whereas previous instantiations of the GODIVA model hypothesised that the neural circuitry for both language (neuron populations for phonological sequences) and motor planning (SSM neuron populations) circuity reside in the left hemisphere, the bilateral GODIVA model makes a different assumption regarding the latter. It posits that for each SSM neuron population (i.e., for each syllable), the majority of neurons are still in the left-hemisphere, but a minority of them are on the right (Fig. 1 in the main text, “RH”). The notion that a minor portion of the speech motor programs represented in GODIVA’s SSM are right-lateralised has both a theoretical and experimental basis. Theoretically, GODIVA places the motor programs at a level that is between a highly left-lateralized frontal language system and a bilateral peripheral motor system. It thus makes sense that the motor program/SSM level is where the transition from left-lateralized to bilateral processing begins. Experimentally, statistically sensitive fMRI studies indicate that right hemisphere homologs of left premotor regions show activity during single word production, albeit substantially less than left hemisphere activity (see Guenther, 2016 for the results of a mega-analysis involving 116 subjects). This adds up to recent functional imaging results and previous clinical studies discussed in Guenther (2016) as well. For the model we use in the simulations, the ratio was approximated to be set specifically to 4:1 (80% left hemispheres, 20% right hemisphere), which is similar to the ratio of left to right hemisphere activity in the Guenther (2016) mega-analysis.

In addition to the bilateral organisation, the model also makes the assumption that each SSM neuron population encoding a syllable is evenly distributed across the whole BA6/44/insula region. This implies higher resilience because a partial lesion of BA6/44/insula will only destroy part rather than the whole neuron population (see hatched slices in Fig. 1 in the main text, for example). The downside, however, is that the neuron populations are now intermingled, and thus, every lesion will affect all syllables to an equal extent.

## Feedback control system: Feedback Control Map and right Articulatory Map

During speech production, the speech system is constantly monitoring speech output, and in the case of inaccuracies (e.g., if there is food within the vocal tract), it generates sensory feedback-based motor commands to correct them. During normal performance, when the activity of a SSM neuron population is supra-threshold and thus the corresponding motor program is read-out normally, feedforward control dominates: the motor program accounts for 85% of the motor output (denoted *α_ff_* = 0.85) (Guenther et al., 2006), whereas feedback-based commands, if required, only accounts for the rest (*α_fb_* = 0.15) (Tourville et al., 2008). However, during atypical speech production,  the weightings of *α_ff_* and *α_fb_* fluctuate. It is also of note that motor planning/execution within the feedback control network is right-hemisphere frontal cortex dominant, involving the Feedback Control Map and right Articulator Map regions of GODIVA/DIVA (Golfinopoulos et al., 2010; Tourville et al., 2008) (Fig. 1 in the main text, righthand).

## Basal ganglia: quenching and activating SSM neuron populations

In the GODIVA model, the shift from one syllable to the next requires quenching the activity of the SSM neuron population for the currently executing syllable (e.g., quenching of “here” in Fig. 1 in the main text). This is because in the model, only one SSM neuron population can have high activity at any point of time as evident in Fig. 5a in the main text (see Bohland et al., 2010 for details on how this winner-takes-it-all behaviour is implemented). When the current syllable loses ground, the next syllable (“is” in Fig. 1 in the main text), which has already primed earlier by input from the IFG, can finally rump up its activity. The quenching should be performed early enough to allow sufficient time to prepare for the shift, i.e., enough time for the activity build-up of the next syllable’s neurons, but on the other hand, not too early such that the current syllable is truncated and not produced in full.

This fine timing of the syllable shifting is controlled in the model by the D2R neurons in the putamen (i.e., striatal projection neurons expressing D2 dopamine receptors). They continuously receive input from ventral motor cortex with a copy of the currently executing motor commands (purely motor program commands, or if feedback control is engaged, their combination with the feedback-based commands, Guenther et al., 2006), and on detecting an articulatory configuration that indicates the imminent completion of the ongoing syllable – usually towards the end of the syllable’s final phone -- they are preset to generate a transient strong inhibitory signal. This signal then performs the quenching of the SSM neuron population (Fig. 5c in the main text) after being transmitted to cortex via the indirect pathway, a pathway that consists of the globus pallidus externa and interna (GPe, GPi), and the thalamus (Kravitz et al., 2010). Although having many functions in the brain, in the current model, these three last regions are merely relays.

As the quenching of the SSM neuron population corresponding to the current syllable gives way to the next syllable, we will refer to the inhibitory signal as the *shift-preparation trigger*. One example for an articulatory configuration that cues a shift-preparation trigger is the rounding of the lips at the phone “o” in “go”; it indicates that the syllable “go” is about to terminate, hence, its SSM neuron population needs to be quenched in preparation for the next syllable. This neural mechanism to shift between syllables is inherited from the extended GODIVA model and is strongly rooted in studies and theoretical consideration of sequencing mechanisms in the brain (see Civier et al., 2013). As neural dynamics in motor cortex were not simulated here, we used simulations in Civier et al. (2013) to algorithmically calculate when the D2R neurons detect the articulatory configuration. Lastly, we assume that the speech system can adjust the sensitivity of this cueing mechanism, making it more strict or lax according to the situation.

The first and last syllables in an utterance are special cases. For the first syllable, there is no previous SSM activity that needs quenching, so the shift-preparation trigger is simply not issued. Instead, the GODIVA model issues a global tonic signal that permits SSM neuron populations to increase their activities (Fig. 5a in the main text, starting from t = 130 ms), and supported by input from the IFG, the population for the first syllable in the utterance reaches threshold first (Bohland et al., 2010). We will not discuss the timing of the last syllable of the utterance as it does not play a part in the effect investigated in the simulations.

## Model components

The brain coordinates associated with the components in the GODIVA/DIVA models are based on studies that were designed specifically in order to localize the neural substrates of speech production (e.g. Bohland & Guenther, 2006; Golfinopoulos et al., 2010; Peeva et al., 2010; Tourville et al., 2008), and as such, they tend to be highly accurate. The coordinates of the cortical regions, which will be studied in the following neuroimaging experiment, are from Guenther (2016) and are given in the Montreal Neurological Institute (MNI) space (Table 1 in the main text, Fig. 2 in the main text). Notice that in addition to BA6/44, the left SSM neuron populations also extend to the adjacent insula, and therefore are assigned to two components (lateral and medial) and two corresponding brain regions (BA6/44 and Insula, respectively). Yet, we assume that both regions have exactly the same functionality in the model. It is also of note that, according to Guenther, the right SSM is limited to the insula region only. Lastly, whereas the left premotor area (BA6) is part of the left SSM lateral, the right premotor is associated with the Feedback Control Map (Golfinopoulos et al., 2010). For consistency, from now on we will refer to the cortical regions using their labels in the GODIVA model.

## REFERENCES

Bohland, J. W., Bullock, D., & Guenther, F. H. (2010). Neural representations and mechanisms for the performance of simple speech sequences. *Journal of Cognitive Neuroscience*, *22*, 1504-1529.

Bohland, J. W., & Guenther, F. H. (2006). An fMRI investigation of syllable sequence production. *Neuroimage*, *32*(2), 821-841.

Brown, J. W., Bullock, D., & Grossberg, S. (2004). How laminar frontal cortex and basal ganglia circuits interact to control planned and reactive saccades. *Neural Networks*, *17*(4), 471-510.

Civier, O., Bullock, D., Max, L., & Guenther, F. H. (2013). Computational modeling of stuttering caused by impairments in a basal ganglia thalamo-cortical circuit involved in syllable selection and initiation. *Brain and Language*, *126*(3), 263-278.

Golfinopoulos, E., Tourville, J. A., & Guenther, F. H. (2010). The integration of large-scale neural network modeling and functional brain imaging in speech motor control. *Neuroimage*, *52*, 862–874.

Grossberg, S. (1978). A theory of human memory: Self-organization and performance of sensory-motor codes, maps, and plans. In R. Rosen & F. Snell (Eds.), *Progress in theoretical biology* (Vol. 5, pp. 233-374). Academic Press.

Guenther, F. H. (2016). *Neural control of speech*. MIT Press.

Guenther, F. H., Ghosh, S. S., & Tourville, J. A. (2006). Neural modeling and imaging of the cortical interactions underlying syllable production. *Brain and Language*, *96*(3), 280-301.

Kravitz, A. V., Freeze, B. S., Parker, P. R., Kay, K., Thwin, M. T., Deisseroth, K., & Kreitzer, A. C. (2010). Regulation of parkinsonian motor behaviours by optogenetic control of basal ganglia circuitry. *Nature*, *466*(7306), 622-626.

Levelt, W. J. M., Roelofs, A., & Meyer, A. S. (1999). A theory of lexical access in speech production. *Behavioral and Brain Sciences*, *22*(1), 1-38.

Peeva, M. G., Guenther, F. H., Tourville, J. A., Nieto-Castanon, A., Anton, J. L., Nazarian, B., & Alario, F. X. (2010). Distinct representations of phonemes, syllables, and supra-syllabic sequences in the speech production network. *Neuroimage*, *50*(2), 626-638.

Tourville, J. A., Reilly, K. J., & Guenther, F. H. (2008). Neural mechanisms underlying auditory feedback control of speech. *Neuroimage*, *39*(3), 1429-1443.
