# Supplementary material for: An Imaging‐Guided Neural Model Explains Lexical Stress Alteration in Acquired Apraxia of Speech
Source: Hum Brain Mapp. 2025 Dec 11;46(17):e70412. doi: 10.1002/hbm.70412 (PMC12696586; doi:10.1002/hbm.70412)
Supplement: Supplementary file 2 — Data S2: hbm70412‐sup‐0002‐supinfo.docx. [file HBM-46-e70412-s002.docx]

| **Participant Identification Code** | |
| --- | --- |
| **Civier et al. (2025)** | **New et al. (2015)** |
| APH_005 | DIS_010 |
| APH_006 | DIS_023 |
| APH_008 | DIS_009 |
| APH_016 | DIS_005 |
| APH_034 | DIS_026 |
| APH_037 | DIS_024 |
| APH_041 | DIS_022 |
| APH_045 | DIS_025 |
| APH_047 | DIS_030 |
| APH_052 | DIS_047 |
| APH_062 | DIS_052 |
| APH_007 | DIS_001 |
| APH_011 | DIS_007 |
| APH_014 | DIS_008 |
| APH_017 | DIS_027 |
| APH_025 | DIS_018 |
|  |  |
| AOS + APH_009 | DIS_002 |
| AOS + APH_018 | DIS_012 |
| AOS + APH_019 | DIS_003 |
| AOS + APH_027 | DIS_011 |
| AOS + APH_030 | DIS_017 |
| AOS + APH_040 | DIS_028 |
| AOS + APH_048 | DIS_029 |
| AOS + APH_055 | DIS_050 |
| AOS + APH_058 | DIS_048 |
| AOS + APH_001 | DIS_014 |
| AOS + APH_010 | DIS_004 |
| AOS + APH_015 | DIS_006 |
| AOS + APH_022 | DIS_015 |
| AOS + APH_043 | DIS_031 |
| AOS + APH_049 | DIS_051 |
|  |  |
